# Evaluation of a social protection policy on tuberculosis treatment outcomes: A prospective cohort study

**DOI:** 10.1371/journal.pmed.1002788

**Published:** 2019-04-30

**Authors:** Karen Klein, Maria Paula Bernachea, Sarah Irribarren, Luz Gibbons, Cristina Chirico, Fernando Rubinstein

**Affiliations:** 1 Institute for Clinical Effectiveness and Health Policy (IECS), Buenos Aires, Argentina; 2 Biobehavioral Nursing and Health Informatics, University of Washington, School of Nursing HSB, Seattle, Washington, United States of America; 3 Tuberculosis Control Program of the 5th Health Region, Ministry of Health of the Province of Buenos Aires, Hospital Cetrángolo, Buenos Aires, Argentina; FIND, SWITZERLAND

## Abstract

**Background:**

Tuberculosis (TB) still represents a major public health problem in Latin America, with low success and high default rates. Poor adherence represents a major threat for TB control and promotes emergence of drug-resistant TB. Expanding social protection programs could have a substantial effect on the global burden of TB; however, there is little evidence to evaluate the outcomes of socioeconomic support interventions. This study evaluated the effect of a conditional cash transfer (CCT) policy on treatment success and default rates in a prospective cohort of socioeconomically disadvantaged patients.

**Methods and findings:**

Data were collected on adult patients with first diagnosis of pulmonary TB starting treatment in public healthcare facilities (HCFs) from 16 health departments with high TB burden in Buenos Aires who were followed until treatment completion or abandonment. The main exposure of interest was the registration to receive the CCT. Other covariates, such as sociodemographic and clinical variables and HCFs’ characteristics usually associated with treatment adherence and outcomes, were also considered in the analysis. We used hierarchical models, propensity score (PS) matching, and inverse probability weighting (IPW) to estimate treatment effects, adjusting for individual and health system confounders. Of 941 patients with known CCT status, 377 registered for the program showed significantly higher success rates (82% versus 69%) and lower default rates (11% versus 20%). After controlling for individual and system characteristics and modality of treatment, odds ratio (OR) for success was 2.9 (95% CI 2, 4.3, *P* < 0.001) and default was 0.36 (95% CI 0.23, 0.57, *P* < 0.001). As this is an observational study evaluating an intervention not randomly assigned, there might be some unmeasured residual confounding. Although it is possible that a small number of patients was not registered into the program because they were deemed not eligible, the majority of patients fulfilled the requirements and were not registered because of different reasons. Since the information on the CCT was collected at the end of the study, we do not know the exact timing for when each patient was registered for the program.

**Conclusions:**

The CCT appears to be a valuable health policy intervention to improve TB treatment outcomes. Incorporating these interventions as established policies may have a considerable effect on the control of TB in similar high-burden areas.

## Abstract 2

Fernando Rubinstein and colleagues evaluate whether a conditional cash transfer policy is associated with improve tuberculosis treatment success for socioeconomically disadvantaged patients in Argentina.

## Author summary

**Why was this study done?:**

- Tuberculosis (TB) still represents a major public health problem in many regions of the world, and although treatment is widely available and highly effective, it is long and burdensome to many patients, leading to suboptimal overall results due to low adherence to treatment.
- Several strategies have been proposed to improve TB treatment adherence using financial incentives to affected individuals and their families, such as conditional cash transfers (CCTs). CTTs can offer a positive incentive to complete TB treatment and hence improve health outcomes, but to date, there is little evidence to evaluate the effectiveness of socioeconomic support interventions in TB.
- This study evaluated the results of a CCT policy on treatment success rates in a group of socioeconomically disadvantaged TB patients in Argentina.

**What did the researchers do and find?:**

- We included 962 patients with a first diagnosis of TB seen in a large number of healthcare clinics and followed them up during the period of their treatment.
- The main results were that those registered for the CCT program showed significantly higher success rates (82% versus 69%) and lower treatment abandonment (11% versus 20%) than those who were not in the program, even taking many other important individual and health system factors into consideration.

**What do these findings mean?:**

- In addition to the availability of a specific treatment, the study suggests that the CCTs may be a valuable health policy intervention to improve the control of TB in similar high-burden areas.
- The results should encourage decision makers to facilitate and promote the implementation of these policies and increase the coverage to all TB patients and households living under vulnerable conditions.

## Introduction

Tuberculosis (TB) is still today a major global public health problem due to its high impact in terms of mortality and morbidity, particularly in economically active groups of low- and middle-income countries.

Despite being a disease with an effective and affordable therapy, treatment success rates are disappointingly low [1] in many settings. A primary cause of low success is due to poor adherence to the challenging treatment regimen that imposes an important burden on patients [2–3]. Several strategies have been proposed to improve TB treatment adherence using financial incentives, such as conditional cash transfers (CCTs). CCTs can offer a positive incentive conditional on a certain behavior, such as an action focused on improving a health outcome [4]. In many countries, CCT programs form the backbone of social security policy as a form of social assistance to improve uptake of health interventions. For TB treatment, CCTs support individuals contingent on taking treatment and attending follow-up appointments. Such a policy offers the advantage of assisting an individual during a critical time when they are required to abstain from work or other activities that could increase the risk of disease transmission to others. Although CCTs are recognized as a potentially powerful tool to promote healthy behaviors, the formal evaluation of the impact of these strategies has been very limited in TB control, particularly in Latin America and the Caribbean. It is important to understand if such programs are effective since they generate costs of implementation and monitoring.

In 1986, the province of Buenos Aires, Argentina, which concentrates 48% of the more than 10,000 notified cases per year in the country, passed a law addressing the use of a CCT as a social support policy to promote adherence to TB treatment for vulnerable patients. To date, only one study reported that patients receiving this financial incentive had more adherence and a higher success rate of treatment [5]. Although an important finding, a next step needed is to consider and assess other potential determinants of TB treatment outcomes, such as access to healthcare, primary source of care, community outreach programs, treatment modality received, comorbidities, and several socioeconomic characteristics. By including and controlling for these potentially confounding variables, we can ascertain a clearer picture of the specific effect of the CCT program on TB outcomes.

### Conceptual framework

CCTs work under the premise that poverty is multidimensional, and modest but regular income from CCTs can help a household smooth consumption and sustain spending on food, household, etc. during the lean period or event (such as required stopping work for treatment) [6]. CCTs can vary in terms of scope (condition to be met or program objectives), benefit structure (cash or in-kind payments, differentiation in payment level), monitoring and enforcement of conditions, and the defined eligible population [7]. The conceptual frameworks presented by Slater and colleagues [8] and Boccia and colleagues [9] may be applicable to guide understanding of interacting influences. Slater argues that there are three principal spheres of impact: institutions, politics, and governance; capacity and implementation; and local economic and social impact. Available resources and potential institutional barriers to uptake of cash transfer should be considered for the institutional, political, and governance sphere. Capacity and implementation involves the capacity of stakeholder, government, and infrastructure. Local economic and social impact entails impact of the cash transfer. The framework also argues that the program should be designed and delivered in a way that beneficiaries recognize and can claim their entitlement (e.g., simple and transparent delivery and accessible information). To better understand the influence of CCTs on TB treatment outcomes, we started by considering other determinants of TB treatment outcomes within these three spheres, such as access to healthcare, primary source of care, availability of community outreach programs, treatment modality prescribed, comorbidities, and several socioeconomic characteristics.

The main purpose of the study was to evaluate the outcomes of different modalities of treatment and a public policy implementing a CCT on treatment outcomes considering a number of specific patient and healthcare system characteristics in a multilevel analysis (MLA). Since the CCT is one of the main health system factors, the main goal of the present paper was to report the effect of this specific intervention in the context of the other characteristics, including the treatment modality prescribed.

## Methods

### Study design and participants

This research was part of a prospective cohort study in 47 healthcare facilities (HCFs) from 16 high-TB–burden health departments reporting a case notification rate (CNR) greater than 60/100,000 in the province Buenos Aires (S1 Text).

After completing a medical history and clinical examination, adult patients (18 years and older) with first diagnosis of pulmonary TB and no known drug resistance initiating treatment were invited to participate and sign the informed consent. The case definition for pulmonary TB was sputum smear‐positive confirmation or diagnosis of pulmonary TB with negative-sputum smear based on radiological findings and clinical signs and symptoms. Exclusion criteria were prior TB treatment, testing positive for drug-resistant TB, and extrapulmonary TB.

### Ethics statement

The study was approved by the Comité de Ética de Protocolos de Investigación Hospital Italiano de Buenos Aires (independent IRB at the Hospital Italiano, Buenos Aires), Argentina (approval #1564), and the Comision Conjunta de Investigación en Salud de la Provincia de Buenos Aires (Central IRB of the Province of Buenos Aires), Argentina (S1 and S2 Approval).

### Procedures

Characteristics of HCFs were collected at baseline. Participants completed a detailed social survey at recruitment, and data on treatment outcomes were collected throughout the course of their follow-up. Patients with first treatment received a 6-month regimen consisting of a 2-month intensive phase of 4 drugs (rifampicin, isoniazid, pyrazinamide and ethambutol, or streptomycin), followed by a 4-month consolidation phase with isoniazid and rifampin daily or 3 times a week [10]. Drug treatment and patient follow-up were provided free of charge in the Public Health System.

Treatment outcomes were evaluated at 2, 4, and 6 months from initiation of treatment by reviewing the TB program assessment card for each participant. There was also a final form once the follow-up was finished (treatment completion, treatment abandonment, transfer, or death). This form also had the necessary information regarding the cash transfer program. Site visits were conducted by trained field assistants who supervised and checked the completion of the patients’ TB cards.

### Variables and outcomes

The main exposure of interest was the registration to receive the cash transfer, defined by the specific elegibility criteria of the law (being a resident of the province of Buenos Aires for at least 2 years and not being covered by any other social security system during the treatment period) [11].

Registration into the program was considered present if the administrative procedures to get the cash transfer were started during treatment (intention to treat) and absent otherwise. Initiation of cash transfer procedures means that the application process was completed by the health professional in charge, who gathered all the required documentation and sent the file to the TB program. The main outcomes, as defined by WHO, were treatment success (cure or completed 6 months) and incomplete TB treatment, defined as the interruption of the treatment for 2 consecutive months or more. If patients stopped treatment for less than 2 months and treatment was reinstated, it was considered an interruption but not a default. Patients who abandoned and were later returned to treatment after 2 months were considered as defaulters.

Other covariates, such as sociodemographic and clinical variables and HCFs’ characteristics usually associated with treatment adherence, were also considered in the analysis. Monthly household income was collected in categories: tercile 1 was less than 245, tercile 2 was 246 to 725, and tercile 3 was more than 725 (US$ 2012).

### Statistical analysis

Since the implementation of the CCT was around 30% of eligible patients, the estimation of the required sample size was determined considering a ratio of no CCT/CCT of 2:1, an estimated incidence of incomplete treatment (default) of 15% in the no CCT group and 8% in the exposed group, a power of 0.80, and a 2-sided alpha error of 0.05. As a result, the estimated required sample size was 472 for the unexposed group and 236 for the exposed group.

We describe and compare the distribution of all covariates between the groups with and without the CCT using tests for continuous and categorical data. Then, crude and adjusted fixed effects were estimated using multilevel logistic regression models [12,13]. MLA has been advocated as a more appropriate statistical method for dealing with outcome data when individual patients are clustered within hospitals or HCFs. The existing standard single-level models, frequently used in outcome studies, treat all patients as independent observations and ignore that characteristics and outcomes of patients treated at the same hospital or HCF may be correlated, violating one of the basic assumptions of traditional regression analysis. MLA also allows the simultaneous examination of the effects of HCF/system-level and individual-level predictors and controls for the nonindependence of observations within groups. Also, MLA is used to estimate the relative contribution of individual- and group-level variables to explain the variability of the outcomes. Thus, MLA allows researchers to deal with the micro level of individuals and the macro level of groups or contexts simultaneously. We assume that both individual factors (age, sex, income, education, employment, alcohol or drug use, comorbidities, etc.) and system factors (treatment modality prescribed, HCF staff and type, community outreach programs, etc.) may be related to TB outcomes, and variables at each level will explain a different proportion of that variability. At the same time, some of those characteristics may also be associated with the probability of registration for the CCT. Therefore, we used an MLA to account for the patient- and system-level characteristics to better estimate the effect of CCT on treatment outcomes.

In addition, since the exposure of interest was an intervention not randomly assigned, we also used propensity score (PS) matching to adjust for group differences and reduce confounding bias [14–16].

The PS is a measure of the probability that an individual is in the “treated” (CCT) group given his or her background (pretreatment) characteristics. Conditional on the PS, it is expected that the distribution of observed baseline covariates will be similar between treated and untreated subjects.

Therefore, we estimated the probability of being registered for the CCT as a function of different variables and used that PS as a matching covariate using nearest neighbor matching. Variables included in the model were age, sex, education, income, drug and alcohol use, employment and marital status, health insurance, source of care, availability of community outreach programs, and treatment modality received: directly observed treatment (DOT), self-administered treatment (SAT), or mixed strategies.

Finally, in order to estimate the treatment effect of this intervention in the context of an observational study, we used IPW regression adjustment (IPWRA) as a strategy for causal inference. All analyses were conducted with STATA 13.

## Results

**Fig 1:**
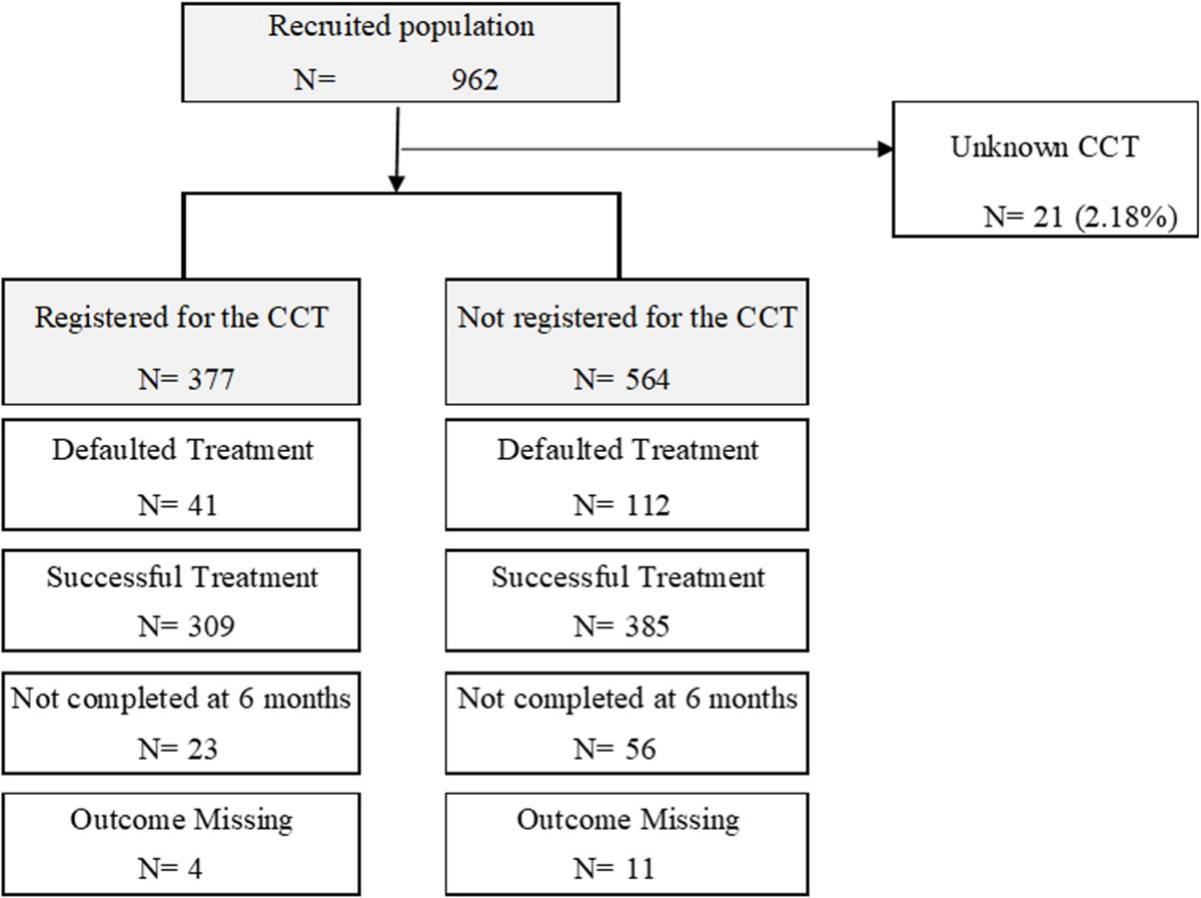
Flow chart.

Recruitment commenced on September 2011, and after reaching the target sample size on June 2014, the last patient follow-up was completed on December 2014. In total, we recruited 962 patients, but in 21, we could not confirm their final group allocation (registered or not to CCT). Thus, of the remaining 941 patients with information on CCT allocation, 377 were registered for the CCT and 564 were not. In each group, there was some information missing on treatment outcome (see Fig 1). Overall, the rate of treatment abandonment was 16.3%. Of the 153 patients who abandoned treatment, 31 did so before completing the second month of treatment, 77 by the fourth month, and 45 by the last visit at 6 months.

Table 1 shows the sociodemographic characteristics and risk factors by group.

**Table 1:** Sociodemographic characteristics and risk factors by group.

Characteristic		Registered for the CCT *N* = 377		Not registered *N* = 564		*P*
		*N*	*n* (%)	*N*	*n* (%)	
**Individual characteristics**		** **	** **	** **	** **	** **
Age*		35.34 (13.67)		35.92 (15.71)		0.566
Females		173	45.89%	258	45.83%	0.985
Nationality	Argentine	334	88.59%	445	78.90%	**0.000**
	Others	43	11.41%	119	21.10%	
Educational level	Elementary	171	46.85%	295	53.54%	0.067
	High School	168	46.03%	211	38.29%	
	Tertiary or university	26	7.12%	45	8.12%	
Live-in couple or married		179	49.72%	260	47.53%	0.518
Currenly working (employed)		136	36.07%	288	51.25%	**0.000**
Type of job	Formal	21	13.82%	148	48.21%	**0.000**
	Informal	132	86.18%	159	51.79%	
Income by tertiles	1	145	39.3%	156	28.21%	**0.000**
	2	175	47.43%	227	41.05%	
	3	49	13.28%	170	30.74%	
Smoking	Current smoker	86	23.12%	118	21.07%	0.459
	Exsmoker or never smoked	286	76.88%	442	78.93%	
Drug use	Current/past drug use	95	45.45%	114	54.55%	0.078
Alcohol use (current or recent)	Yes	86	22.87%	120	21.43%	0.601
Distance to the healthcare center	Less than 1 km	152	40.43%	224	39.86%	0.862
	More than 1 km	224	59.57%	338	60.14%	
Health coverage (private or social security)	Yes	35	9.28%	204	36.17%	**0.000**
HIV test		219	58.09%	346	61.57%	0.286
HIV positive^		19	8.72%	41	11.95%	0.142
Treatment strategy^&^	SAT	235	62.33%	410	72.6%	**0.002**
	DOT	89	23.61%	87	15.43%	
	Mixed	53	14.06%	67	11.88%	
**Health center variables**						
Hospital based#		164	43.5%	307	54.43%	**0.001**
Social worker		352	93.62%	534	95.02%	0.358
Community programs		205	54.38%	336	59.57%	0.114
TB program training		222	59.68%	349	64.51%	0.138
TB program supervision		230	61.99%	381	70.56%	**0.007**
Successful treatment		309	82.6%	385	69.5%	**0.001**
Abandoned treatment		41	10.99%	112	20.25%	**0.000**
Death		1		4		

**Abbreviations:** CCT, conditional cash transfer; DOT, directly observed treatment; SAT, self-administered treatment; TB, tuberculosis.

*Continuous variables are expressed in mean and standard deviation.

^ Among those who did the test.

# Versus primary care center based treatment.

& SAT (reference category).

Mixed: DOT at least 2 months and SAT the remaining 4 months.

*P* < 0.05 is considered statistically significant. *P* < 0.05 in bold font.

Crude analysis shows that being registered for the program was strongly associated with treatment success and default rates, with those not under the CCT showing significantly lower success rates (69% versus 83%) and higher default rates (20% versus 11%), respectively, both *P* < 0.001.

As expected, there were several differences among those included and not registered in the CCT program. Registered patients were more commonly not employed or had an informal job, lower income, and lack of health insurance. DOT or mixed treatment modality was more prevalent than SAT as well as having a primary care center as their main source of care. Age, gender, marital status, educational level, smoking, drug use, HIV status, distance to healthcare center, and other variables related to healthcare were not associated with being or not being registered in the program.

In addition to the CCT program, other individual factors significantly associated with higher default rates were nationality, alcohol and drug use, smoking, type of job, lowest income tertile, and lack of health insurance. Younger patients also showed a higher risk of abandonment. Treatment modality was strongly associated with success and default rate, with the SAT group showing significantly higher default rates than those receiving DOT or mixed regimes (20.3% versus 8.3% and 7.5%), respectively.

Regarding system-level variables, receiving care at a hospital versus a primary care center, lack of community outreach programs and lack of periodic supervision from the TB program also were related with higher default rates.

We used a multilevel logistic regression model to estimate the adjusted effect of the program on default and success rates, adjusting for all of the identified potential confounders at the individual and the healthcare system level.

As seen in Table 2, compared to patients not registered in the program (reference category), the crude odds ratio (OR) for abandonment for those registered was 0.45 (95% CI 0.30, 0.68, *P* < 0.001), suggesting a significantly lower risk in this group.

**Table 2:** Multilevel model. Factors associated to incomplete treatment (default).

Characteristic		Crude OR	95% CI	Adjusted OR		95% CI	*P* value
**Cash Transfer **		**0.45**	**0.30**–**0.68**	**0.36**		**0.23–0.57**	**0.000**
Treatment^&^	DOT	0.36	0.20–0.63	0.45		0.23–0.88	**0.020**
	Mixed	0.32	0.16–0.65	0.31		0.14–0.70	**0.004**
Male		1.35	0.95–1.92		0.84	0.54–1.30	0.44
Age		0.98	0.97–0.99	0.97		0.96–0.99	**0.002**
Currently employed		1.22	0.86–1.73	1.15		0.77–1.72	0.501
Live-in couple or married		0.94	0.66–1.33	1.22		0.82–1.82	0.331
Educational level	High School	1.05	0.73–1.5	0.89		0.58–1.39	0.618
	Terciary or university	0.44	0.16–1.06	0.48		0.19–1.21	0.118
Income tertiles*	2	0.57	0.38–0.84	0.70		0.43–1.15	0.162
	3	0.49	0.30–0.80	0.52		0.27–0.99	**0.049**
Smoking		1.64	1.11–2.42	1.09		0.66–1.84	0.717
Drug user		2.45	1.69–3.56	1.59		0.99–2.51	**0.050**
Alcohol abuse		2.24	1.53–3.27	1.68		1.05–2.67	**0.029**
Primary care center versus hospital		0.56	0.39–0.80	0.72		0.42–1.24	0.230
Community programs		0.60	0.42–0.85	0.74		0.42–1.30	0.294
TB program supervision		0.44	0.31–0.63	0.77		0.39–1.51	0.460

**Abbreviations:** DOT, directly observed treatment; SAT, self-administered treatment; TB, tuberculosis.

& SAT (reference category).

Mixed: DOT at least 2 months and SAT the remaining 4 months.

*Tertile 1 reference category.

*P* < 0.05 is considered statistically significant. *P* < 0.05 in bold font.

Variables used for adjustment: level 1 (individuals): sex, age, employment and civil status, education, income, use of alcohol or drugs, and smoking. Level 2 (health system): treatment modality, source of care, availability of community programs, and regular TB program supervision of the center

### Multilevel models

The variability in default rates was substantial among the different HCFs. The intracluster correlation coefficient (ICC) was significant (ICC 0.123 [95% CI 0.08–0.17]), meaning that although the individual patient characteristics explain most of the variability, approximately 12% of the total variance can be explained by characteristics of the system and HCFs (level 2) (primary clinic- versus hospital-based care, CCT, community outreach, training of healthcare teams, treatment modality prescribed, etc.) The multivariable model in Tables 2 and 3 show that, after adjusting for the most important individual and health system factors, being registered for the CCT was associated with a substantially lower odds of default and higher odds of success: adjusted ORs 0.36 [95% CI 0.23, 0.57], and OR 2.9 [95% CI 2, 4.3] respectively, both *P* < 0.01. Other variables associated with a higher risk of incomplete treatment were SAT, younger age, lack of insurance, lower income, and use of alcohol and illicit drugs.

**Table 3:** Multilevel model: Factors associated to treatment success.

Characteristic		Crude OR	95% CI	Adjusted OR	95% CI	*P* value
**Cash Transfer **		**2.08**	**1.49–2.92**	**2.91**	**1.97–4.28**	**0.001**
Treatment^&^	DOT	2.40	1.53–3.77	1.82	1.06–3.17	**0.029**
	Mixed	2.25	1.34–3.77	1.92	1.08–3.47	**0.031**
Male		0.74	0.55–1.01	1.03	0.71–1.49	0.820
Age		1.02	1.01–1.03	1.01	1.00–1.03	**0.030**
Currently employed		0.90	0.67–1.22	1.16	0.81–1.68	0.410
Live-in couple or married		0.94	0.69–1.26	0.78	0.55–1.18	0.160
Educational level	High School	1.16	0.85–1.58	1.06	0.73–1.56	0.770
	Tertiary or university	1.87	0.97–3.59	1.61	0.77–3.37	0.200
Income tertiles*	2	1.23	0.87–1.74	0.95	0.64–1.42	0.883
	3	1.18	0.79–1.76	1.01	0.61–1.65	0.970
Smoking		0.57	0.41–0.80	0.76	0.49–1.13	0.251
Drug user		0.42	0.30–0.58	0.54	0.36–0.81	0.003
Alcohol abuse		0.54	0.38–0.75	0.68	0·45–1·03	0·070
Primary care center versus hospital		1.95	1.44–2.64	1.44	1.03–2.08	0.048
Community programs		1.57	1.17–2.12	1.18	0.70–1.99	0.570
TB program supervision		2.18	1.60–2.97	1.82	1.05–3.14	**0.03**

**Abbreviations:** DOT, directly observed treatment; SAT, self-administered treatment; TB, tuberculosis.

& SAT (reference category).

Mixed: DOT at least 2 months and SAT the remaining 4 months.

*P* < 0.05 is considered statistically significant. *P* < 0.05 in bold font.

*Lower tertile as reference

Variables used for adjustment: level 1 (individuals): sex, age, employment and civil status, education, income, use of alcohol or drugs, and smoking. Level 2 (health system): treatment modality, source of care, availability of community programs, and regular TB program supervision of the center.

Since this was a prospective cohort study and to facilitate interpretability of main results, we used the adjusted OR obtained from the multilevel logistic regression to estimate the corresponding adjusted relative risks (formula in S2 Text) [17]. Hence, the adjusted relative risk (RR) for successful treatment in patients in the CCT group was 1.25 (1.18, 1.31) and for incomplete treatment was 0.41 (0.27, 0.62), both *P* < 0.001.

The PS matching yielded a good balance among the variables used for adjustment (see Table 4). The region of common support for PS matching was between 0.15 and 0.72. Individuals in non overlapping areas of the PS were not considered in the matched analysis (49 controls and 31 in CCT original groups).

**Table 4:** Comparison of patient characteristics between CCT and control groups before and after PS matching.

Variable	Before PS matching			After PS matching		
	CCT (*n* = 377)	Control (*n* = 564)	*P* value	CCT (*n* = 346)	Control (*n* = 515)	*P* value
**SAT**	62.33%	72.69%	0.001	62.71%	66.47%	0.302
**Sex**	54.11%	54.17%	0.985	54.62%	56.35%	0.647
**Years of education**	8.31	8.22	0.679	8.23	8.29	0.796
**Age in years**	35.34	35.91	0.566	34.83	34.66	0.880
**Alcohol**	22.87%	21.42%	0.602	22.83%	24.85%	0.533
**Addict**	25.19%	20.32	0.079	26.01%	26.59%	0.863
**Employed**	36.07%	51.24%	0.000	36.41%	36.41%	1.000
**HCF**	56.49%	45.56%	0.001	56.64%	58.09%	0.701
**Terciles of income**	39.30%	28.21%	0.000	39.03%	41.34%	0.564
	47.43%	41.05%	0.056	47.39%	45.95%	0.673
	13.28%	30.74%	0.000	13.58%	12.71%	0.782

**Abbreviations:** CCT, conditional cash transfer; HCF, healthcare facility; PS, propensity score; SAT, self-administered treatment.

Using PS matching and IPWRA as alternative methods to adjust for confounders yielded essentially the same results as the multilevel models, with estimated adjusted treatment effects of an absolute reduction of abandonment of −12.6% 95% CI (−7.7%, −17.5%) and an absolute increase in treatment success rate of 15% 95% CI (9%, 21%), both *P* < 0.001 (S2 Text).

## Discussion

Our results show that patients registered for the CCT had greater success rates and were less than half as likely to have incomplete treatment after controlling for individual and healthcare system factors as potential confounders.This suggests that the registration for this financial incentive (i.e., the intent to grant this CCT) had a significant effect on adherence to TB treatment, independently of age, educational and income level, employment and marital status, source of care, availability of community programs, and modality of treatment received. Interestingly, the exposed group could nearly achieve the WHO goal of at least 85% completed treatment [18].

Although TB care is provided free of charge by the public system, patients incur important direct expenses to access the treatment and may lose or reduce their source of income if they cannot work. This program intends to provide social protection and promote treatment completion among TB patients. It is made effective through the payment of a monthly amount, which today represents a percentage of the minimum wage category of the public administration in the province, to all eligible patients identified and incorporated into the Provincial TB Control Program (PTP).

Beneficiaries are mandated to keep health controls, treatments, and other conditions established by the PTP; failure to do so may result in the loss of the benefit. The process needs to be initiated by a health professional. A social worker and a physician evaluate each case, taking into account the severity, the socioeconomic situation, the community risks, and the most susceptible age groups. Once the candidate is individualized, they advise him to complete the application form and the relevant obligations. When this process is completed, the social worker and the attending physician state in the same request that the patient is eligible for the program. This request must be attached to the social report and the medical record and sent to the PTP, who is responsible for evaluating the application and granting or denying the requested subsidy. This is a long, complex, and bureaucratic process that generally lasts more than the duration of the treatment, and at times, the patients receive the benefit up until 1 year after completion. Thus, unfortunately, this “TB-specific” intervention has not been broadly available to patients in the province.

Another systematic review and meta-analysis assessed the effects of social protection on TB treatment outcomes in low- or middle-income and in high-burden countries [19]. This review included 12 studies evaluating financial interventions (within them, only one retrospective cohort evaluating a CCT [20] and one randomized clinical trial (RCT) evaluating a monetary incentive with vouchers) [21]. The other RCTs evaluated food incentives [22,23] and social support or educational interventions [24–29]. This systematic review showed that social protection was associated with TB treatment success, cure of TB patients, and reduction in risk of incomplete treatment (TB treatment default). However, the authors also concluded that overall quality of evidences regarding these effect estimates is low. Therefore, there is limited evidence to support that sustained incentive programs can improve long-term adherence to TB treatment. Most of the evaluated interventions were isolated incentives and not a formal social protection policy program, as is the case in our study, which has existed for more than 20 years but has not yet been widely implemented.

Some observational studies were also conducted. A review of the impact of cash transfer and microfinance interventions for TB control [4] found that these interventions have the potential to improve people’s access to TB services and reduce people’s vulnerability to TB by improving households’ socioeconomic situations. However, given the relatively short follow-up period in many of the studies, little can be concluded concerning the sustainability of these findings. This study concluded that synergies between social protection interventions and TB control programs could be effective. A cross-national statistical modeling analysis performed in 21 European countries [30] showed that an increase in social protection spending was associated with a decrease in the number of TB case notifications, estimated incidence rates, and mortality rates, but no association was found on smear-positive treatment success. Another observational study performed in the city of New York [31] showed that the odds that a patient would adhere to therapy were greater with increased incentives, independently of clinical, demographic, or social factors.

Several other studies with different methodologies provided similar insights supporting the use of cash transfer programs [32–38]. However, important methodological issues in the published studies (small sample size, contextual factors affecting the intervention, interventions conducted in traditionally hard-to-reach or marginal populations, lack of adequate adjustments, etc.) might limit the evaluation of the potential effect of this strategy to reduce abandonment of TB treatment.

One recent RCT [39] evaluated the impact of a social support program and a CCT on prevention and treatment success in shantytowns in Peru. It showed that treatment was successful in 64% (87/135) of patients receiving the socioeconomic support versus 53% (78/147) in the control arm.

As mentioned before, our study is an observational study evaluating an intervention not randomly assigned. Hence, some selection bias may play a role since the two compared groups have differences in certain characteristics associated with the outcomes, and there might be some unmeasured residual confounding not accounted for by this analysis. However, we used rigorous methodologies to control for confounders and for site and population selection. The consistent results of the multilevel logistic regression, the PS approach, and the causal inference analysis used to adjust for multiple factors suggest a significant effect of the CCT program on treatment success and abandonment. Also, the study population was drawn from broad heterogeneous healthcare settings that included semirural and urban settings that likely have similarities to other public healthcare systems in Latin American or other low- and middle-income countries (LMICs).

In 2014, there were 9,600 notifications of new cases in Argentina; of those, almost 8,000 were pulmonary TB and 90% of them older than 15 years. Approximately 30% of these cases comes from the departments included in the study. Therefore, approximately 2,200 cases a year could be potentially eligible, around 6,600 during the accrual period (3 years). We enrolled 962 patients, around 15% of the estimated eligible population. We have not transferred this information to the flow chart because we do not have the exact number of potential eligible populations for the departments included nor the exact number of those who were invited or declined to participate.

Although only 20% of CCT-registered patients started to receive the transfers during the treatment period, the remaining 80% received it within the 12-month period following the completion. Interestingly, the benefit of the CCT was evident even with this important delay, reinforcing the potential effect of the program.

It is possible that a small number of patients was not registered into the program because they were deemed not eligible by the center health professional. However, the vast majority of patients not enrolled fulfilled the requirements but did not get registered due to different reasons, such as lack of awareness from health providers and patients about the CCT program or complex registration process and bureaucratic barriers. Since the information on the CCT was collected at the end of the study, we do not know the exact timing for when each patient was registered for the program. Nevertheless, all patients included in the program received the total amount corresponding to the full 6-month treatment period independently of the date of the registration.

The threat of multidrug-resistant TB (MDR-TB) continues to spread. It was recently estimated that in 2017, there were about 558,000 people developing TB resistant to rifampicin, of whom an estimated 458,000 had MDR-TB, defined as resistance to two first-line drugs, rifampicin and isoniazid, and 230,000 deaths globally. Argentina is one of the 5 countries in the Americas with a high number of estimated MDR-TB [40]. The main cause is poor adherence to TB treatment, and the consequences in public health are enormous: second-line drugs are less effective and treatments are much longer and substantially more expensive with more adverse effects, leading to a vicious circle of higher default rates and potentially catastrophic consequences [34]. Identifying interventions to improve compliance and reduce abandonment of treatment may represent a great contribution to reduce MDR-TB.

Sustainable Development Goal 1 (SDG 1) focuses on reducing poverty and expanding social protection. The link between poverty and TB has been well described, and evidence from ecological studies supports an association between increased social protection and decreased TB burden [35].

A recent study estimated the reduction in global TB incidence that could be obtained by reaching SDG 1’s targets of reducing poverty and expanding social protection. The model suggested that expanding social protection coverage may result in a reduction in TB incidence of 76% by 2035 [36]. These results align with the concept of Syndemics, an interesting approach to study, understand, and implement health research [37], refining the conventional frameworks that overlook the effects of social, political, and ecological factors and illuminating how macro-level social factors impact on health. As stated recently [38], purely biomedical or public health solutions are not enough to end the TB epidemic; countries must implement social policy strategies that can protect the patients and their contexts and prevent incomplete treatment since these interventions represent a critical and necessary investment.

We performed a formal evaluation of a health policy that aims to benefit not only individuals but also families and communities. We believe that our study provides valuable quantitative evidence of the effect of a CCT on sustained TB treatment adherence to project its effects and additional groundwork to support this strategy. The CCT appears to be a valuable health policy intervention to improve the control of TB in similar high-burden areas. The results of this study should encourage decision-makers to facilitate and promote a much wider implementation of these policies and increase the coverage to all TB patients and households living under vulnerable conditions.

## Supporting information

S1 STROBE STROBE checklist. STROBE, Strengthening the Reporting of Observations Studies in Epidemiology.
(PDF)

S1 Text Design and analysis. (PDF)

S2 Text PS and IPW adjustment. IPW, inverse probability weighting; PS, propensity score.
(PDF)

S1 Approval IRB approval: Hospital Italiano. IRB, Institutional Review Board.
(PDF)

S2 Approval IRB approval: Province of Buenos Aires. IRB, Institutional Review Board.
(PDF)

S1 Appendix CRF patient form. CRF, case report form.
(PDF)

## Abbreviations

CCT: conditional cash transfer
CNR: case notification rate
DOT: directly observed treatment
HCF: healthcare facility
ICC: intracluster correlation coefficient
IPW: inverse probability weighting
IPWRA: IPW regression adjustment
LMIC: low- and middle-income countries
MDR-TB: multidrug-resistant TB
MLA: multilevel analysis
OR: odds ratio
PS: propensity score
PTP: Provincial TB Control Program
RCT: randomized clinical trial
RR: relative risk
SDG 1: Sustainable Development Goal 1
SAT: self-administered treatment
TB: tuberculosis
