# Correction: Evaluation of a social protection policy on tuberculosis treatment outcomes: A prospective cohort study

**DOI:** 10.1371/journal.pmed.1002826

**Published:** 2019-05-31

**Authors:** Karen Klein, Maria Paula Bernachea, Sarah Iribarren, Luz Gibbons, Cristina Chirico, Fernando Rubinstein

The third author’s name is spelled incorrectly. The correct author name is Sarah Iribarren. The affiliation should read: Biobehavioral Nursing and Health Informatics, University of Washington, Seattle, Washington, United States of America.

The citation should read: Klein K, Bernachea MP, Iribarren S, Gibbons L, Chirico C, Rubinstein F (2019) Evaluation of a social protection policy on tuberculosis treatment outcomes: A prospective cohort study. PLoS Med 16(4): e1002788. https://doi.org/10.1371/journal.pmed.1002788
