# Supplementary material for: Evaluation of a social protection policy on tuberculosis treatment outcomes: A prospective cohort study
Source: PLoS Med. 2019 Apr 30;16(4):e1002788. doi: 10.1371/journal.pmed.1002788 (PMC6490910; doi:10.1371/journal.pmed.1002788)
Supplement: S1 STROBE — STROBE, Strengthening the Reporting of Observations Studies in Epidemiology. (PDF) [file pmed.1002788.s001.pdf]

STROBE Statement—Checklist of items that should be included in reports of ***cohort studies***			
	**Item No**	**Recommendation**	
**Title and abstract**	1	(*a*) Indicate the study's design with a commonly used term in the title or the abstract	Title: Evaluation of a social protection policy on tuberculosis treatment outcomes: A prospective cohort study.
		(*b*) Provide in the abstract an informative and balanced summary of what was done and what was found	The abstract provides this required information.
**Introduction**			
Background/rationale	2	Explain the scientific background and rationale for the investigation being reported	The Introduction explains the scientific background and rationale for the investigation
Objectives	3	State specific objectives, including any prespecified hypotheses	Objectives are stated in the last two paragraph of the Introduction.
**Methods**			
Study design	4	Present key elements of study design early in the paper	The study design is stated in the Title, the Abstract and the first paragraph of Methods.
Setting	5	Describe the setting, locations, and relevant dates, including periods of recruitment, exposure, follow-up, and data collection	Setting and location is specified in paragraph 1 of the *Methods section*. The dates of data collection are described in paragraph 1 of the Results section<br>Periods of recruitment, exposure and follow-up are explained in the *Procedure section* in paragraph 4 of the Methods.
Participants	6	(*a*) Give the eligibility criteria, and the sources and methods of selection of participants. Describe methods of follow-up	The section *Study design and participants* gives this information in paragraph 1 of Methods.
		(*b*) For matched studies, give matching criteria and number of exposed and unexposed	Number of exposed and unexposed and variable balance achieved by the Propensity score matching are included in paragraph 10 and 11 of Results.

Variables	7	Clearly define all outcomes, exposures, predictors, potential confounders, and effect modifiers. Give diagnostic criteria, if applicable	The section *Variable and outcomes* gives these definitions in paragraph 6, 7 and 8 of the Methods section.
Data sources/ measurement	8*	For each variable of interest, give sources of data and details of methods of assessment (measurement). Describe comparability of assessment methods if there is more than one group	Explained in paragraph 5 of Methods under the section *Procedures*.
Bias	9	Describe any efforts to address potential sources of bias	In the section *Statistical Analysis* we explained how we addressed potential bias (paragraph 11 of Methods)
Study size	10	Explain how the study size was arrived at	In paragraph 9 of Methods, under the *Statistical Analysis* we explained how the study size was arrived.
Quantitative variables	11	Explain how quantitative variables were handled in the analyses. If applicable, describe which groupings were chosen and why	The detailed analyses are explained in the section *Statistical Analysis*.
Statistical methods	12	(*a*) Describe all statistical methods, including those used to control for confounding	The detailed analyses are explained in the section *Statistical Analysis*.<br><br>Please also see S1 Propensity Score & IPW adjustment.
		(*b*) Describe any methods used to examine subgroups and interactions	Please also see S1 Propensity Score & IPW adjustment.
		(*c*) Explain how missing data were addressed	We had a few patients with missing information on the exposure (2.18%) and outcomes (1,6%). They were not included in the analysis. See Fig 1: Flow chart
		(*d*) If applicable, explain how loss to follow-up was addressed	Patients with missing outcome information were not included in the analysis
		(*e*) Describe any sensitivity analyses	Not applicable
**Results**			
Participants	13*	(a) Report numbers of individuals at each stage of study—eg numbers potentially eligible, examined	Please see Fig 1 (flow chart) and the explanations in the Results section (paragraph 1)

		for eligibility, confirmed eligible, included in the study, completing follow-up, and analysed	
		(b) Give reasons for non-participation at each stage	Please see Fig 1 (flow chart) and the explanations in the *Results section* (paragraph 1)
		(c) Consider use of a flow diagram	Please see Fig 1.
Descriptive data	14*	(a) Give characteristics of study participants (eg demographic, clinical, social) and information on exposures and potential confounders	Please sse Table 1.
		(b) Indicate number of participants with missing data for each variable of interest	Please See Fig 1.
		(c) Summarise follow-up time (eg, average and total amount)	Follw-up for our study was 6 months. See paragraph 5 in Methods.
Outcome data	15*	Report numbers of outcome events or summary measures over time	Not applicable
Main results	16	(*a*) Give unadjusted estimates and, if applicable, confounder-adjusted estimates and their precision (eg, 95% confidence interval). Make clear which confounders were adjusted for and why they were included	Explained in tables and Results section.
		(*b*) Report category boundaries when continuous variables were categorized	Not applicable
		(*c*) If relevant, consider translating estimates of relative risk into absolute risk for a meaningful time period	Estimated adjusted treatment effects are reported from paragraph 8 in Results section.
Other analyses	17	Report other analyses done—eg analyses of subgroups and interactions, and sensitivity analyses	Pleases see *Statistical analysis* and supplementary material (S1 Propensity Score & IPW adjustment).
**Discussion**			

Key results	18	Summarise key results with reference to study objectives	First paragraph of the Discussion.
Limitations	19	Discuss limitations of the study, taking into account sources of potential bias or imprecision. Discuss both direction and magnitude of any potential bias	In the Discussion section (paragraph 9)
Interpretation	20	Give a cautious overall interpretation of results considering objectives, limitations, multiplicity of analyses, results from similar studies, and other relevant evidence	All this items are described in the *Discussion section*.
Generalisability	21	Discuss the generalisability (external validity) of the study results	Last paragraph of Discussion section.
**Other information**			
Funding	22	Give the source of funding and the role of the funders for the present study and, if applicable, for the original study on which the present article is based	Please see *Role of the funding source*.
