# Supplementary material for: Evaluation of a social protection policy on tuberculosis treatment outcomes: A prospective cohort study
Source: PLoS Med. 2019 Apr 30;16(4):e1002788. doi: 10.1371/journal.pmed.1002788 (PMC6490910; doi:10.1371/journal.pmed.1002788)
Supplement: S1 Text — (PDF) [file pmed.1002788.s002.pdf]

PI: **rubinstein, fernando adrian**	Title: Patient and system factors associated with successful treatment of Tuberculosis	
Received: 09/18/2009	FOA: PAR08-130	Council: 05/2010
Competition ID: ADOBE-FORMS-A	FOA Title: International Research in Infectious Diseases including AIDS (IRIDA) Program (R01)	
**1 R01 AI083229-01A1**	Dual:	Accession Number: 3225569
IPF: 10008655	Organization: INSTITUTO DE EFECTIVIDAD CLINICA Y SANIT	
Former Number:	Department:	
IRG/SRG: ZRG1 IDM-R (50)R	AIDS: N	Expedited: N
Subtotal Direct Costs<br>(excludes consortium F&A)<br>Year 1: 94,100<br>Year 2: 100,930<br>Year 3: 86,970	Animals: N<br>Humans: Y<br>Clinical Trial: N<br>Exemption: E7<br>HESC: N	New Investigator: Y
Senior/Key Personnel:	*Organization:*	*Role Category:*
FERNANDO RUBINSTEIN M.D.	Instituto de Efectividad Cl??nica y Sanitaria (IECS)	PD/PI
MARIA CHIRICO MD	Ministry of Health, Province of Buenos Aires	Other Professional-ASSOCIATE INVESTIGATOR
LUZ GIBBONS Sr.	INSTITUTE FOR CLINICAL EFFECTIVENNESS AND HEALTH POLICY	Other Professional-Biostatistician
DAVID MOORE	Universidad Peruana Cayetano Heredia	Other Professional-CONSULTANT
Richard Oberhelman	TULANE UNIVERSITY	Other Professional-CONSULTANT
JUAN PALMERO	INSTITUTE FOR CLINICAL EFFECTIVENNES AND HEALTH POLICY	MPI
VIVIANA RODRIGUEZ	INSTITUTE FOR CLINICAL EFFECTIVENNESS AND HEALTH POLICY	Other Professional-Researcher in Infectious Diseases

A. SPECIFIC AIMS

Tuberculosis (TB) is a leading cause of morbidity and death worldwide and remains a major health problem in Latin America and Argentina, especially in a number of high burden areas [3,-7]. The province of Buenos Aires concentrates 48% of the more than 10,000 notified cases per year in the country and implements direct observation of treatment (DOT) in only 37% of the detected cases [2]. Success (cure or completion of treatment) and default rates ranged from 53-66% and 12-20% between 1998 to 2007 [8] when recommendations from the WHO Stop TB program is to successfully treat 85% of diagnosed cases [8].

DOT is a component of the internationally recommended strategy for TB control (Directly Observed Therapy Short course, or DOTS) [9]. The effectiveness of DOT to improve treatment outcomes has generated recent debate and controversy [10-13]. In Argentina, there are few studies assessing TB in general [2, 3, 14-17], fewer evaluating strategies [1, 3, 7], and no studies systematically evaluating other factors affecting treatment success.

The **purpose** of this study is to assess the effectiveness of treatment strategies, as well as identify and assess the impact of patient and health care facility (HCF) factors on treatment outcomes in a cohort of patients with newly diagnosed pulmonary TB in high burden departments of Buenos Aires, Argentina.

The **main hypothesis** to be tested is that after controlling for individual and health care facility factors (Table 1)*,* treatment success will be *significantly greater* and default rate will be *significantly lower* in patients assigned to *DOT* compared with those assigned to *self administered therapy* (*SAT).* A **secondary hypothesis** is that, aside from DOT, we can identify specific patient and health care facility characteristics associated with treatment success (high and low risk subgroups) that can be used to support professional decision-making for treatment regimens and to optimize resource allocation. In order to approach the proposed objectives of the study we propose to carry out this cohort study with a multilevel multilevel analytic approach.

A.1 Study Aims

1. To evaluate the associations between patient and HCF characteristics with treatment outcomes

1.1. To evaluate the effect of each factor on treatment outcomes

1.2. To identify factors, *distinct* from DOT, significantly associated with treatment outcomes

2. To evaluate selected patient and HCF-specific factors to estimate the *adjusted* effects of DOT on treatment outcomes

2.1. To evaluate the adjusted effect of DOT when other confounders are controlled for in a multilevel regression model.

2.2. To estimate the proportion of variability of treatment outcomes attributable to characteristics of the patient and the health care facility

We will prospectively follow a sample of approximately 900 patients with pulmonary TB starting treatment in 35 to 40 different HCF in high burden departments of Buenos Aires. A multilevel model (hierarchical analysis) will be used to estimate the adjusted effect of DOT and other identified factors on treatment outcomes, as well as estimate the proportion of variability in the outcomes attributable to the different levels.

3. To explore a potential different effect of supervised treatment (DOT versus SAT) on treatment success within groups defined by *high* and *low* risk profiles identified in multi-level analysis.

4. To identify modifiable factors that can be applied to interventional studies to optimize resource allocations and impact policy.

[39].

Figure 2. Framework for Study

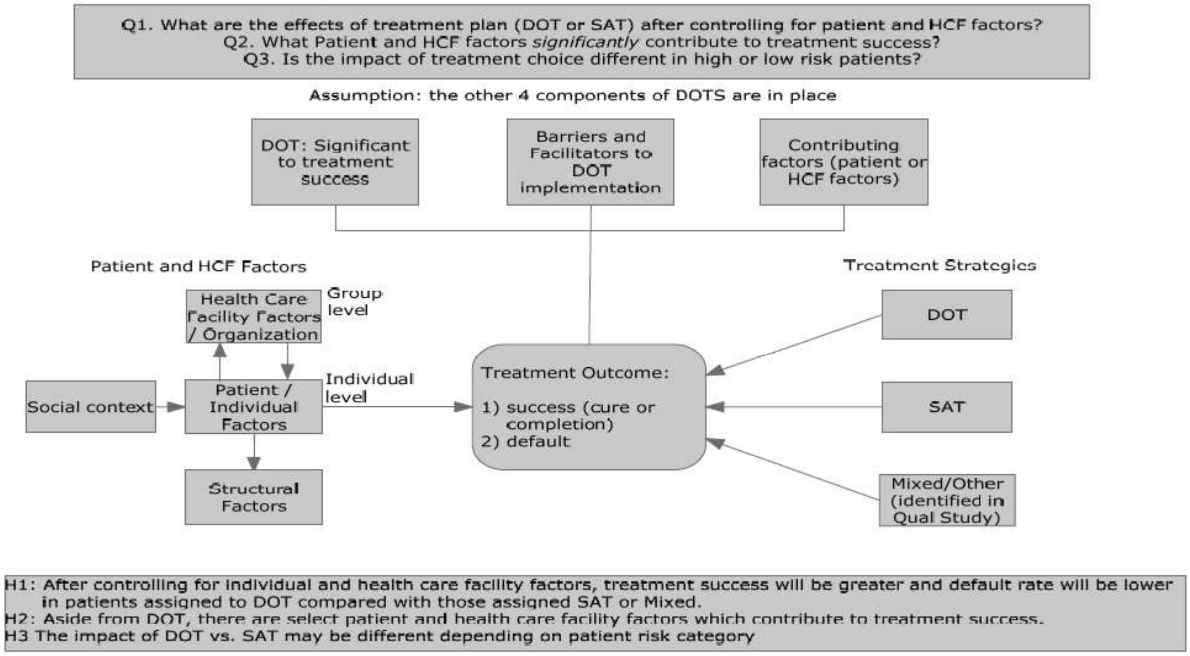

Patient and health care facilities adapted from [34]
HCF: health care facility, DOT: direct observation of therapy. SAT: self administration of therapy

B.2 Research Design

Mixed method studies can provide an in-depth understanding of the phenomenon by capitalizing on the strengths of both research paradigms [41, 42]. Qualitative research approaches the topic through exploration, induction and hypothesis generation, while quantitative research focuses on deduction, confirmation and hypothesis testing using statistical analysis [41]. [The first phase of this study focused on an exploration of barriers in a local context (see preliminary studies and [43].]
The present proposal will be a systematic hypothesis driven evaluation of the variables by applying quantitative methods. Public health policy research is increasingly using multilevel analysis to look at variations across different levels simultaneously [44]. Multilevel analysis (MLA) or hierarchical models have been advocated as a more appropriate statistical method for dealing with outcomes data when individual patients are clustered within hospitals or health care facilities [45]. The MLA framework will allow the partitioning of the total variation in successful treatment into factors attributed to the patient and to HCF, and will provide flexibility to explore the specific characteristics or factors at each level that may explain the differences in treatment outcome. The process of MLA will serve to elucidate the effect of treatment strategies more clearly within the patient and HCF context [46].

B.3 Treatment strategies

The evaluation of treatment strategies is possible because the other four critical components of the DOTS strategy: government commitment to sustain the program, quality laboratory services, uninterrupted effective anti-tuberculosis drug supply and a monitor and evaluation system to assess treatment effects for each patient and provide information on overall TB management [22, 47] are currently in place [14, 48].
In a Cochrane systematic review synthesizing the evidence from ten randomized and quasi-randomized controlled trials, with a total of 5609 participants, comparing DOT and SAT on treatment success found no statistically significant differences in cure or treatment completion between the two arms (RR 1.02, 95% CI 0.86 to 1.21, random

effects model, 4 studies), calling into question DOT component [27]. However, the findings are challenged by advocates of DOT who counter that the studies cited reported lower than expected cure and success rates in both arms of the studies, suggesting that direct observation may not have been implemented effectively [24]. A recent well conducted cluster randomized trial that recruited 1522 patients showed a significant benefit from DOT compared to SAT both in success and default rates, 88% (RR 1.18, CI 1.03-1.34) in intervention group and 76% in control group, default was 5.5.% versus 16.8% (RR 0.43, CI 0.21-0.89) [36]. Interestingly, success rates for both groups were much higher than those reported by the trials included in the Cochrane review [27].

It is recognized that treatment success hinges on a multitude of challenging, dynamic and complex factors [23, 34]. The aim is not to enter into the debate of merely DOT versus SAT or non-DOT, but rather to take into account the complex structure of multiple factors at both the individual and public health level simultaneously that result in treatment outcomes. The actual application of DOT ranges throughout the world from inpatient hospitalization for treatment to outpatient settings where health care workers, trained community or family member can be observers of TB treatment and assist in managing the drugs [10-13, 23, 26]. The current recommendation is for DOT to be flexible and adapted to the setting, facility, resources and environment and to the needs of the patients and conditions of health care workers to maximize treatment outcomes [21].

Subsequently, the adapted treatment method needs to be evaluated for effectiveness on success rates (both completion and default) to serve as either a model or to be modified if it does not achieve the expected results [21]. The NTP of Argentina documents results as supervised and SAT, anecdotal evidence suggests that treatment plans aside from DOT or SAT are currently implemented in Argentina. Qualitative inquiry served to define the predominant strategies that are in place in addition to exploring the barriers and facilitators to the application of the currently recommended DOT.

B.4 Patient and Health Care Facility Factors

Researchers in qualitative studies have sought to determine factors that are associated with treatment adherence [11, 44, 45]. However, there has been the tendency to focus on patient-related factors when considering the challenging and complex treatment adherence rather than health care system or structural factors [23]. Multiple factors may account for the success of treatment [23, 34]. Primary patient factors to be assessed will include: socio-demographic information, distance to travel to HCF, patient knowledge, treatment strategy and social support (see table 3, design and method section for full description of variables). Health care facility factors will include: staff turnover rate, use of incentive, treatment strategy, case load, and presence of community-based programs. If significant modifiable factors are identified, the aim is to develop approaches that optimize both patient and HCF needs and can guide and support professional decision-making for treatment regimens that also optimize resource allocation.

In summary, a representative sample of patients taken from high burden departments throughout the province of highest TB patient population will strengthen the understanding factors related to treatment outcomes, as well as factors to explain treatment course. Moreover, studies investigating treatment success tend to evaluate treatment strategies retrospectively or prospectively with patients divided into treatment groups [26] without controlling for individual or HCF factors that may significantly contribute to the treatment outcome. Because of the complexity of treatment success in a given population, this study proposes a design that will prospectively assess factors and predictors, which traditionally have been studied in cross-sectional studies. Building on the design, theoretical framework (figure 2) and multilevel statistical methodology will lead to a more powerful understanding of treatment strategies and factors across a time span. Thus, the purpose of this study is to examine treatment strategies controlling for patient and HCF factors and also identify modifiable factors that can be used to establishment of a platform for further interventional studies aiming to improve treatment success in Argentina.

D. RESEARCH DESIGN AND METHODS

D.1 Overview

The **objectives** of this study in high TB burden departments in the province of Buenos Aires, Argentina are to:

(1) understand ***barriers and facilitators*** for the implementation of directly observed treatment (DOT);

(2) estimate the ***adjusted effect of treatment plan*** (DOT or Self administered treatment (SAT)) and ***patient and health care facility (HCF) characteristics*** on ***treatment outcomes*** (success and default rate) (Table 1); and

(3) evaluate specific patient and HCF ***characteristics associated with treatment success*** in order to identify risk subgroups that can be used to both support professional decision-making for treatment regimens and to optimize resource allocation.

*[The study design is built upon a sequential **mixed-method (Qualitative / Quantitative)** design [35]. Emphasis of the present proposal will be placed on the cohort study using a multilevel analysis to assess the impact of the different treatment modalities and identify specific patient and HCF factors that influence outcomes.*

The qualitative data obtained from the literature and our own study (see C.3.c Preliminary Studies Conducted by study team members) will supplement quantitative analysis, providing a richer foundation for understanding barriers and facilitators and other specific factors that are considered by patients and health care providers, which cannot be fully understood through the traditional quantitative methodology. A sequential mixed-methods approach provides a strong foundation for systematic evaluation, while providing in-depth focus to explore and identify potential testable factors associated with treatment success.]

The *first phase* using qualitative research techniques (in-depth interviews and focus groups) with patients and health providers explored and identified testable and non-testable barriers and facilitators for the implementation of DOT; and actual treatment strategies being applied in HCF that are not within the established primary strategies (e.g. DOT or SAT). Major themes identified in qualitative study will be integrated with established variables from the literature to guide this quantitative phase of the study (Table 3, below).

This proposed study, the *quantitative phase,* is an observational cohort study that will follow patients with newly diagnosed pulmonary TB initiating treatment at select health care facilities (HCF) throughout the duration of their treatment. The **dependent variables** are treatment outcomes (treatment success and treatment default, see table 1 for definition of terms), while the **independent variables** are patient and HCF factors (Table 3, below) and treatment plan (e.g. DOT or SAT). The data collected in the observational study will be used to evaluate quantitative objectives using a multilevel analytic approach (MLA) (see section A, Study Aims).

D.2 Study Design

The study design is based on an **Exploratory – Explanatory Qualitative / Quantitative)** mixed method design [35]. The study proceeds as a primary exploration of contextual variables which will complement the subsequent hypothesis driven quantitative study design and analysis. Mixed methods research integrates traditional quantitative and qualitative research paradigms into a combined design, maximizing the strengths of both research paradigms [35]. It represents a pragmatic approach to obtaining valuable answers to questions best responded by different methodological frameworks [36]. Traditional quantitative research focuses on deduction, confirmation and hypothesis testing using statistical analysis, whereas qualitative research approaches the topic through exploration, induction and hypothesis generation [35]. Thus, combined methods are complementary and will provide robust methodology to explore and expand our knowledge of barriers and facilitators to DOT implementation and systematically evaluate factors

and strategies influencing treatment outcomes. Ultimately, the goal of using mixed method research strategy is to capitalize on both methods to address each research aim and inform future interventional studies aiming to improve treatment success in high burden departments of Buenos Aires, Argentina. Results may also be applicable to urban settings with low treatment success rates and low implementation of DOT strategy.

D.2.a Cohort Study

Departments with high burden of TB are defined by the established criteria from the Argentina's National Tuberculosis Program (NTP) (Table 1). HCFs will be selected within those departments and include all patients fitting the inclusion criteria, see D.3.b. The estimated sample size is 1100 patients, see table 2, *Sample size calculation based on cluster size and ICC.* Patients participating in the study will be those initiating first treatment for pulmonary TB at selected facilities from the period [*January 1, 2011 through July 31, 2012, (until recruitment of estimated sample size).*] Data to be collected will include participating patients and HCF factors (Table 3) collected from the National TB card and questionnaire. The cohort of patients will be followed until [*December 31, 2012*,] allowing time for the last patient included in the study to complete treatment.

A multilevel regression model (hierarchical analysis) will be used to estimate the adjusted effect of DOT and specific factors on the treatment outcomes, as well as estimate the proportion of variability in the outcomes attributable to the different levels (patient and HCF). Multivariable analysis will also be used to identify subgroups with different risk for treatment outcomes (Table 4). The identified significant modifiable factors (e.g. need for decentralization of TB program, patient education, provider training, turn-over of health care personnel, use of incentives, etc) may provide essential information for the design of adapted interventions for future investigation.

D.2.b Rationale for the use of Multilevel Analysis

Multilevel analysis (MLA) has been advocated as a more appropriate statistical method for dealing with outcomes data when individual patients are clustered within hospitals or health care facilities [39]. The existing standard single-level models, frequently used in outcome studies, treat all patients as independent observations and ignore that characteristics and outcomes of patients treated at the same hospital or health care facility may be correlated, violating one of the basic assumptions of traditional regression analysis [39].

Multilevel analysis allows:

(a) the simultaneous examination of the effects of group-level and individual-level predictors;

(b) the non independence of observations within groups is accounted for since groups or contexts are not treated as unrelated, but are seen as coming from a larger population of groups; and (d) the analysis of both inter-individual and inter-group variation (as well as the contributions of individual level and group level variables to these variations) [40]. Thus, multilevel analysis allows researchers to deal with the micro level of individuals and the macro-level of groups or contexts simultaneously, explaining variation in the dependent variable at one level as a function of variables defined at various levels, plus possible interactions within and between levels. Multilevel analysis is one methodology that can be used to approximate the latter situation [40].

We assume that in addition to the treatment plan assigned, the variability in treatment success is based on variation across individuals and across health care facilities; suggesting that each level will explain a different proportion of that variability. See Figure 1 for model of multilevel analyses.

The following are hypothetical examples to illustrate the **analytic approach** of MLA in this study. Individual patient characteristics may explain some of the variation; e.g. women may have higher success rates than men; success may increase with age and may be highest among highest educated individuals. Another example is that some HCF may treat more patients with lower SES. At the same time, some of those characteristics may be associated with receiving DOT or SAT.

Therefore, adjusting for the patient level characteristics may correct the magnitude of the crude association of DOT with treatment outcomes. Likewise, we will use characteristics or factors at the HCF level to help explain differences in success rates among HCFs. For example, we may consider if a HCF uses a community outreach program or a unique treatment strategy may be more successful than others. Again, in order to understand the patient and HCF factors, the initial qualitative phase was crucial.

The MLA framework will allow us to separate or partition the total variation in successful treatment into factors attributed to the patient and to HCF, and also provides flexibility to explore the specific characteristics or factors at each level that may explain the differences in outcome. The process of MLA will serve to elucidate the effect of treatment strategies more clearly within the patient and HCF context. Ultimately, if the different levels are not considered, there is a risk that too many relationships will be found to be "significant" [38].

The outcome will be measured at the patient level (treatment success or default) but it is a joint product of both individual and HCF level factors. Three different types of hypotheses are possible with MLA [38]. First, that the individual characteristics are responsible for outcome rather than compositional effects. That is, a community that is attended by a specific HCF is composed of patients with the same characteristic rather than an actual or *contextual* effect of the HCF. Second, if there is a contextual effect at the level of the HCF, then hypotheses can be tested about the relationship among different HCF factors. For example, the implementation of the different treatment strategies (DOT, SAT or other identified strategy), taking the individual patient characteristics into account. Lastly, cross-level interactions can be assessed. An example, which will be evaluated in the study, will be the possible finding of a different effect of DOT on treatment outcomes in patients defined as low or high risk of success or default based on specific individual and HCF factors. Success rate is an indicator of the quality of the NTP however, in order to accurately measure performance, treatment strategies should be adjusted for patient and HCF level factors.

Figure 1. Health Care Facility and Patient level model

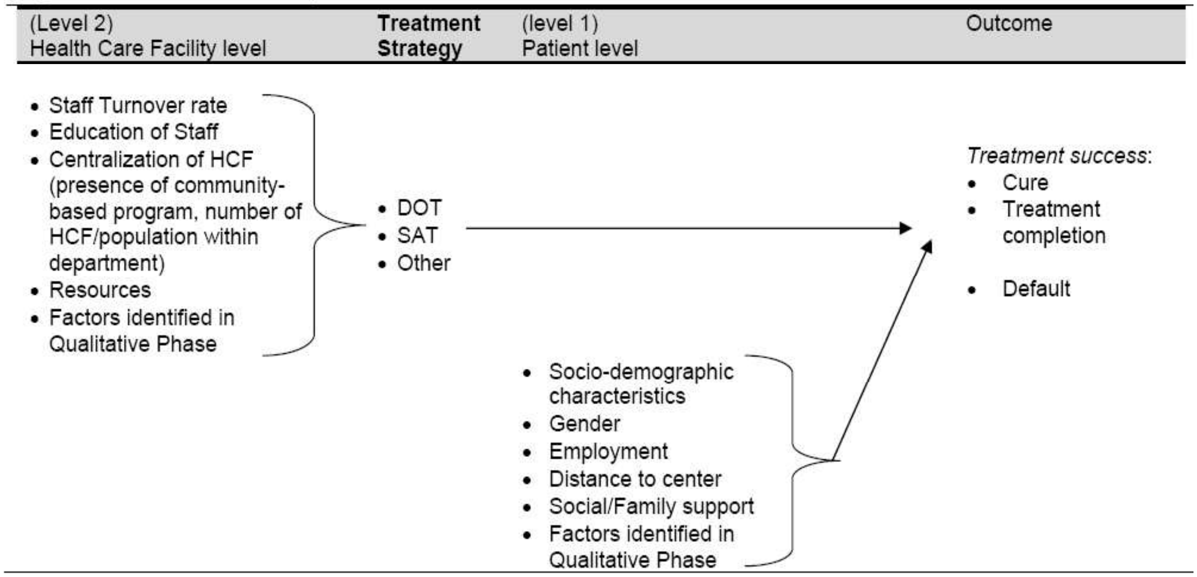

Table 1. Terms with Definitions

Term	*Definition*
**DOTS**	Strategy comprised of 5 components; (1) *government commitment* to sustain program, (2) *case detection* through quality laboratory sputum testing, (3) *standardized treatment* with direct observation therapy (DOT) with course of 6-8 months of treatment, (4) uninterrupted *effective anti-tuberculosis drug supply,* and (5) a monitoring and evaluation system to assess treatment effects for each patient and provide information on overall TB management.
**DOT**	Direct observation of treatment (medication ingestion) by a trained treatment supporter. One component of DOTS strategy. Recommended for at least the first two months of the intensive phase of anti-tuberculosis treatment.
**SAT**	Self administration of treatment, not directly observed.
**Treatment success**	Cure or completion of the treatment<br>Cure: confirmed by negative sputum at smear at 6 months and on at least 1 previous occasion<br>Completion: full treatment completion without bacteriological confirmation.
**Default**	Patient who interrupted treatment for at least two months
**Percent of cases evaluated**	Percent of patients reported to be sputum smear-positive for whom there is follow-up information available in the monitoring system.
**Transferred out**	Patients transferred to other facilities for whom there is no further information
**High TB burden department**	Annual case notification rate (CNR) > 75th percentile or number of cases reported/year > 95th percentile for the country or the region.

Adapted from Treatment of tuberculosis: Guidelines for tuberculosis [46] and National tuberculosis program of Argentina [42]

D.3 COMPONENTS FOR THE COHORT STUDY

D.3.a Setting

The province of Buenos Aires is comprised of 138 departments that are accountable for both public and private HCFs within each jurisdiction. The study will be conducted in select HCFs within high burden departments in the province, defined in table 1. The province is located in the center-east region of Argentina, it is considered the most important province of the country due to its size (307.571 km^2), population and vital economic activities for the country. The total population of the province is approximately 18 million inhabitants of which 13.3 million reside in the great Buenos Aires region, which surrounds the capital district area. The population under 15 years is 25.4%, over 65 years 10,3%, illiteracy rate 1,6%, unemployment rate 9,6% and 26,6% are below the poverty line [47].

Tuberculosis diagnosis and treatment are provided by basic health services. Treatment, care and medication, is provided free of cost in the Public Health System. Consistent with WHO recommendations [9], first time TB diagnosed patients receive a 6-month treatment regimen consisting of a two month *intensive* phase of four drugs (rifampicin, isoniazid, pyrazinamide, and ethambutol or streptomycin), followed by a four month *continuation / consolidation* phase with two drugs (isoniazid and rifampin) daily or intermittently (3 times a week) [42]. Drug provision and patient monitoring are the responsibility of the NTP. At a minimum, WHO recommends that patients receive DOT during the intense phase to maximize treatment adherence [9].

[The HCFs that will be included in this study are public primary care centers and hospitals from the Province of Buenos Aires. Based on the organization of the Public health care system, municipal hospitals coordinate a variable number of primary care centers distributed in the community. These centers have the essential medical services; physician, nursing, social workers and some have dentists, psychologists, basic radiology and laboratory services. Each Primary care centre has a geographical area of influence but does not have a unique or defined population to care for. Hospitals are secondary or tertiary centers, with different levels of complexity and services in relation to their role in the system and location (i.e. Municipal, Regional,

Provincial). Attendance at the hospitals is free of charge and does not require previous authorization or referral from lower care levels.

The bacteriological diagnosis of patients with TB is performed at laboratories from the public network working with the NTP. Samples collected at Primary care centers without availability of lab services are transported to central laboratories or patients are referred to them in order to obtain the sputum samples. According to a recent survey of bacteriological diagnosis of TB in Argentina [14], smear and culture testing centers are being provided at or above the WHO recommended density. The percentage of smear negatives among all bacteriologically confirmed pulmonary cases was 18.9%, while most extrapulmonary cases were confirmed by culture. The microscopy quality assessment program covered most high-demand laboratories in Argentina.]

D.3.b Inclusion/Exclusion Criteria: Selection of health care facilities and patients

• ***Inclusion Criteria for Departments*****:** High burden departments will be selected by using the NTP database of cases for the province of Buenos Aires. High burden departments are defined by the NTP as those reporting a case notification rate (CNR); (a) equal or superior to the 75th percentile and / or; (b) reported cases greater than the 95th percentile for the province. In 2006 there were over 30 departments that met this definition.

• ***Inclusion Criteria for Health Care Facilities*****:** Facilities treating an average of 25 pulmonary TB patients (lowest limit 15) within the previous year. If the number of facilities available is greater than the number needed for the study, participating facilities will be randomly selected among them

• Patients

- ***Case definition***

Patients in category I or III as defined by the Guidelines of the National TB Program. New case of pulmonary TB confirmed by sputum smear-positive or diagnosis of pulmonary TB, based on radiological findings and clinical signs and symptoms but with negative sputum smear. In these cases the diagnosis may be confirmed by methods other than sputum smear such as MGIT960, BACTEC 9000 or MB Bact, nucleic acid amplification (PCR) or ELISA.]

- ***Patient inclusion criteria*****:**

(a) first diagnosis of pulmonary TB, enrolled at initiation of therapy;

(b) 18 years or older; and

(c) non drug resistant TB, if tested.

- ***Patient exclusion criteria*****:**

a) prior TB treatment; b) testing positive for drug resistant TB when unconfirmed resistance status at treatment initiation; and c) diagnosis of extra-pulmonary TB

D.3.c Sample recruitment and retention

To maximize recruitment and retention, coordination and cooperation with the health care facilities personnel and the officers of the TB program at each department will be essential. At the onset of study preparation, the study will be presented to all program managers and selected centers. The purpose of the meetings will be to present the study, promote participation, and train health care providers for the enrollment of patients. The health care team members at each participating HCF will: 1) discuss the nature of the study with patients (2) obtain consent; (3) administer initial questionnaires including factors, defined in table 3; and (4) collect standard information for the patient TB card. HCFs will be compensated for each patient interview at a rate of $15USD, equivalent to the cost of a visit.

D.3.d Site Permissions and Consents

Approval and support for the study has been obtained from the Director of the National Tuberculosis Control Program (See letter of support). Department Officers of the TB program at each department, as well as directors of participating health care facilities will be asked to sign a permission form. Written informed consent for all participating patients will be obtained at recruitment.

D.4 Cohort Study

D.4.a Sample

Sample will include all consenting patients meeting study criteria being treated for pulmonary TB seen at selected centers. [*Recruitment period will be from January 1, 2011 through July 31, 2012, (or until completing the estimated sample size). Patients will be followed until December 31, 2012, allowing time for the last patient included in the study to complete the recommended 6 months treatment.*]

D.4.b Study Procedure

Patients will be followed throughout the course of their treatment regimen. The research team will be responsible for monitoring the recruitment, registration, treatment, and follow-up of patients. Formal monitoring visits to the participating HCF will be conducted by recruited and trained field assistants, who will supervise and check the completion of the patients' TB cards, following the procedures of the NTP. Treatment outcomes will be measured using standard definitions set by the WHO Standards of TB treatment (Table 1). Primary outcomes of interest will be both the proportion of recruited patients experiencing treatment success (those patients cured and those completing their full course of treatment) and those defaulting from treatment. For the present study, patients who default and are returned to treatment will be defined as defaulters.

D.4.c Power Analysis.

Based on previous studies, treatment success rates among patients receiving DOT versus those under SAT have been approximately 85% and 70% respectively [1, 9, 26, 31]. Because patients will be grouped within health care facilities, we expect some degree of intra-cluster correlation represented by an intra-class correlation coefficient *[(ICC) ranging from 0.10 to 0.15. With an average cluster size of 25 to 30 patients we will need to include between 32 and 43]* health care facilities to detect a 15% difference with 80% power and an error type I probability of 5% (Table 2).

Table 2. Sample size calculation based on cluster size and ICC

ICC	Number of clusters needed			
	N = 25	N = 30	N = 35	N = 40
0.01	12	10	9	8
0.03	16	15	14	13
0.05	21	19	18	17
0.07	25	24	23	22
0.10	32	31	30	29
0.15	43	42	41	40
0.20	55	53	52	52

D.4.d Instruments

Patient identification and general information will be obtained from a personal interview at recruitment and from the patient TB treatment card. Follow up information will be obtained from the patients' TB treatment cards kept at the health centers throughout the duration of the treatment period. HCF level information will be obtained from a standard instrument of the NTP, the Health

Care Facility evaluation form based on the WHO Survey instrument and guide to implementation (Available at *www.**anlis**.gov.ar/acrobat/normare3.pdf, page 135-157)*. A supplemental instrument for patients and HCF data is anticipated from the results of the first phase of the study to accompany the prior mentioned instruments.

Table 3. Independent variables: Patient and Health Care Facility Definitions and source

Data from survey instrument	
Patient Level Factors	*Health Care Facility Factors*
Socio-demographic characteristic	*Health Care Facility of treatment*
Age	*[Hospital, primary health care center,]*
Gender	*[Public or Private]*
Marital Status [stable partner, single, divorced, widowed]	*[Urban / non-urban]*
Employment status [Employed/Unemployed]	
Educational level [none, primary, secondary, tertiary or university]	
Income [ranges]	
Socioeconomic status [categories by National Surveys]	
Housing [type of housing by National Surveys]	
Distance to the center [km]	*Overall daily case load per health professional [not exclusively TB patients]*
Transportation mode [walk, bus, train, drive self, friend/family member]	
Co-morbidities (chronic diseases) [List]	
Tobacco consumption [current, past, never]	
HIV status [positive, negative, not known]	
Family support/ Social support [presence or absence of support network]	
Treatment regimen [DOT, SAT, Other]	
Treatment assignment [6 or 8 month regimen, daily or 3x/week]	

Illicit Drug use (any type) [current, ever, not ever]	
Data from literature[29,39] and own qualitative study	
Information on TB [questionnaire]	
Bond with the center and health care staff	*Network organization of TB care [coordinating center, laboratory on site services vs referral]*
Difficulties for reemployment [yes / no]	*Availability of anti-TB drugs [yes / no]*
Poverty and social exclusion [above or below poverty/indigence line]	*Information management system [availability of computers vs paper form based tracking system]*
Presence of community leaders [yes / no]	*Political support [yes / no]*
Data from follow-up study	
Report of side effects of treatment during follow-up [yes / no]	*Staff turn-over rate [permanence of key team members for at least two years]*
Data from WHO NPT evaluation Questionnaire	
	Specific TB training activities of the health care team
	Presence of Community-based programs [yes / no]
	Percentage of patients under DOTS coverage [Facility DOTS coverage]

Data from ad hoc survey instrument	
Patient Level Factors	***Health Care Facility Factors***
Socio-demographic characteristic	***Health Care Facility of treatment***
Age	*[Hospital, primary health care center,]*
Gender	*[Public or Private]*
***Marital Status** [stable partner, single, divorced, widowed]*	*[Urban / non-urban]*
***Employment status** [Employed/Unemployed]*	
***Educational level** [none, primary, secondary, tertiary or university]*	
***Income** [ranges]*	

Socioeconomic status *[categories by National Surveys]*	
Housing *[type of housing by National Surveys]*	
Distance to the center *[km]*	***Overall daily case load*** *per health professional [not exclusively TB patients]*
Transportation mode *[walk, bus, train, drive self, friend/family member]*	
Co-morbidities (chronic diseases) *[List]*	
Tobacco consumption *[current, past, never]*	
HIV status *[positive, negative, not known]*	
Family support/ Social support *[presence or absence of support network]*	
Treatment regimen *[DOT, SAT, Other]*	
Treatment assignment *[6 or 8 month regimen, daily or 3x/week]*	
Illicit Drug use (any type) *[current, ever, not ever]*	
Data from the literature and our qualitative study	
Information on TB *[questionnaire]*	
Bond with the center and health care staff	***Network organization of TB care [coordinating center, laboratory on site services vs referral]***
Difficulties for reemployment [yes / no]	***Availability of anti-TB drugs [yes / no]***
Poverty and social exclusion [above or below poverty/indigence line]	***Information management system [availability of computers vs paper form based tracking system]***
Presence of community leaders [yes / no]	***Political support [yes / no]***
Data from follow-up study	
Report of side effects of treatment during follow-up [yes / no]	***Staff turn-over rate*** *[permanence of key*
	team members for at least two years]
Data from WHO NPT evaluation Questionnaire	
	Specific TB training activities of the

	health care team
	Presence of Community-based programs *[yes / no]*
	Percentage of patients under DOTS coverage *[Facility DOTS coverage]*

D.4.e DATA COLLECTION PROCEDURES

Data Collection. Data from HCF will be collected at baseline. Monitoring visits to the participating HCF will be conducted monthly by recruited and trained research assistants. Data for patients will be collected at baseline and every two months thereafter until completing treatment. Therefore, data will be collected for six months after the last patient is enrolled in the study. At recruitment, in addition to the information collected on the patient TB card, initial questionnaires including factors defined in table 3 will be administered to all consenting patients by members of the health care team at the participating HCF. Follow up data (treatment course) will be obtained from the TB card and patient record. At the end of the follow up period for each patient. Final outcomes will be ascertained as success (cure or completion), default, transferred out, dead or other. Research team members will make attempts to trace and contact all enrolled patients for whom definitive outcome information is not available at the HCF in order to determine final status.

A questionnaire evaluating the capacity and routine practices of HCF seeing TB patients will be administered as part of the regular evaluation of the NTP. Questionnaires for data collection of patient information will be in a format similar to the TB card. The questionnaire will be pre-tested for one month in a center not participating in the study. The data collected will not be different from the prior data collected for routine patient care.

Data Management: entry and quality control. We will use data collection forms specific to the study. Data collectors will be trained field assistants, who will supervise and check the completion of the patients' TB cards, supplemental questionnaires and health care facility data, following the procedures of the NTP. All forms will be recorded onto standardized Case Report Forms. The supervisor will keep the original data forms and the data collector will keep copies.

The data manager at IECS will train the data collectors. All forms will be reviewed by trained staff prior to data entry. All fields will be reviewed for illegal or out of range codes. If missing fields or illegal values are encountered, the data manager / supervisor will review the original source data with the data abstractor and make corrections as appropriate. Impossible, inconsistent and invalid data will be identified and corrected.

Data will be double-entered into a Microsoft Access or similar database. This double entry will be used to ensure validity. The inconsistencies identified by the validation process will be entered into a logbook. A query sheet will be produced and submitted to the data supervisor who will use other available sources of information to correct the data. The modifications will be entered in the database and in the logbook.

Special care will be taken to ensure that study materials with patients' identification information are stored securely in a location that differs from that of the remaining study data.

D.5 PLANNED ANALYSIS.

We will prospectively follow a sample of approximately 900 to 1000 patients with pulmonary TB starting treatment in 35 to 40 different HCF in high burden departments of Buenos Aires. In addition to standard multiple logistic regression models a multilevel analytic approach (hierarchical analysis)

will be used to estimate the adjusted effect of DOT and other identified factors on treatment outcomes, as well as estimate the proportion of variability in the outcomes attributable to the different levels.

Hypotheses

H1: After controlling for individual and health care facility factors (Tables 1 & 3), *treatment success* will be greater and *default rate* will be lower in patients assigned to *DOT* compared with those assigned to SAT.

H2: Aside from DOT, there are select *patient* and *health care facility* factors which contribute significantly to treatment success

Table 4. Hypotheses, Variables and Analysis

Hypothesis	Variables	Analysis
DOT improves outcomes independently of other individual or system factors	DOT vs. SAT<br>Other patient and HCF factors	MLR<br>HLM
Socio-demographic and other individual characteristics of patients are related to outcomes	Age, Gender, Marital Status, N° living in household, Employment status, Educational level, SES, Family support/ Social support<br>Co-morbidities, illicit drug use	MLR<br>HLM
Patient knowledge of TB improves outcomes	Pt knowledge evaluated in questionnaires	MLR<br>HLM
Decentralization of HCF improves treatment outcomes	• Distance and transportation<br>• Primary Health care facilities vs. hospital<br>• Community based activities	MLR<br>HLM
Staff frequent turnover is associated with worse outcomes	Stable staff = Proportion of HCF staff on site for more than 2 years	MLR<br>HLM

MLR: Multiple logistic regression. HLM: Hierarchical linear model

Study Aims

1. To evaluate the associations between patient and HCF characteristics with treatment outcomes (successful completion of treatment / cure or default)

1.1. To evaluate the effect of each factor on treatment outcomes

1.2. To identify factors, *distinct* from DOT, significantly associated with treatment outcomes

Analyses:

a) General Description of patients and health facilities: Means and 95% Confidence intervals or medians and inter quartile ranges will be reported for continuous variables. Proportions will be reported for the categorical variables.

b) Evaluation of the association of the patient and center level characteristics and treatment modality with treatment success and default rate. We will use standard test for continuous variables; t test for independent samples or Wilcoxon rank sum tests depending on the data distribution and chi square tests for proportions for the categorical variables. In addition, we will report crude odds ratios and 95% confidence intervals (CI) for factors associated with treatment outcomes.

2. To evaluate selected patient and HCF-specific factors to estimate the *adjusted* effects of DOT on treatment outcomes

2.1. To evaluate the adjusted effect of DOT when other confounders are controlled for in a multilevel regression model.

2.2. To estimate the proportion of variability of treatment outcomes attributable to characteristics of the patient and the the health care facility

3. To explore a potential different effect of supervised treatment (DOT versus SAT) on treatment success within groups defined by *high* and *low* risk profiles identified in multi-level analysis.

4. To identify modifiable factors that can be applied to interventional studies to optimize resource allocations and impact policy.

Analyses: A Multilevel analysis on two levels (patients and health care facilities) will be performed. Effects of all significant variables will be reported as adjusted odds ratios and 95% CI.

a) We assume that the data obtained have a nested or hierarchical structure, that is, individuals (patients) are nested within groups (health centers), thus considering two levels of nesting.

b) Data will be analyzed by multilevel regression, treating patients as the unit of analysis, and taking into account the dependence of outcomes of patients nested within the same health center. We consider that the distinction between group-level and individual-level variables becomes relevant in this study since variables that reflect some specific characteristics of different centers are assumed to capture group properties that are more than summaries of individual properties.

c) When effect in a statistical model is modelled as being random, we mean that we want to draw conclusions about the population from which the observed units were drawn, rather than about these particular units themselves. In this sense, we will apply random effects modelling in both levels considered.

d) By incorporating multiple levels of determination in the study of individual outcomes, multilevel analysis will allow us to control the effects of group- and individual-level variables as well as their interactions.

Regression equations will be formulated for each level (patient, center) of the multilevel data structure, and will be then combined into a single equation. Covariates to be measured at the individual-level and at the group-level are described in table 3.

e) Based on the adjusted effect of DOT and other potentially modifiable factors we will estimate the expected impact of each variable and each level on treatment outcomes using an attributable risk proportion approach.

Data-Sharing Plan

Annual reports to NIH will be prepared and submitted. Results of the study will be presented to directors of the HCF and NTP program officers of each department. A final report will be prepared for the NTP.

Presentations on study findings are anticipated at the national and international level. Manuscripts will be prepared and submitted for publication.
