# Supplementary material for: Evaluation of a social protection policy on tuberculosis treatment outcomes: A prospective cohort study
Source: PLoS Med. 2019 Apr 30;16(4):e1002788. doi: 10.1371/journal.pmed.1002788 (PMC6490910; doi:10.1371/journal.pmed.1002788)
Supplement: S2 Text — IPW, inverse probability weighting; PS, propensity score. (PDF) [file pmed.1002788.s003.pdf]

SUPPLEMENTARY MATERIAL

PROPENSITY SCORE

EVALUATION OF COVARIATE BALANCE BETWEEN CCT AND CONTROL GROUP

Table 4: Comparison of Patients characteristics between Conditional Cash Transfer (CCT) and Control groups before and after Propensity Score (PS) Matching.

Variable	**Before PS Matching**				**After PS Matching**			
	**CCT (n=377)**	**Control (n=564)**	**p Value**		**CCT (n=346)**	**Control (n=515)**	**p Value**	
SAT	62.33%	72.69%	0.001		62.71%	66.47%	0.302	
Sex	54.11%	54.17%	0.985		54.62%	56.35%	0.647	
Years of education	8.31	8.22	0.679		8.23	8.29	0.796	
Age in years	35.34	35.91	0.566		34.83	34.66	0.880	
Alcohol	22.87%	21.42%	0.602		22.83%	24.85%	0.533	
Adict	25.19%	20.32	0.079		26.01%	26.59%	0.863	
Employed	36.07%	51.24%	0.000		36.41%	36.41%	1.000	
HCF	56.49%	45.56%	0.001		56.64%	58.09%	0.701	
Terciles of income	39.30%<br>47.43%<br>13.28%	28.21%<br>41.05%<br>30.74%	0.000<br>0.056<br>0.000		39.03%<br>47.39%<br>13.58%	41.34%<br>45.95%<br>12.71%	0.564<br>0.673<br>0.782	

Graph 1. Standardized % bias before and after PS matching

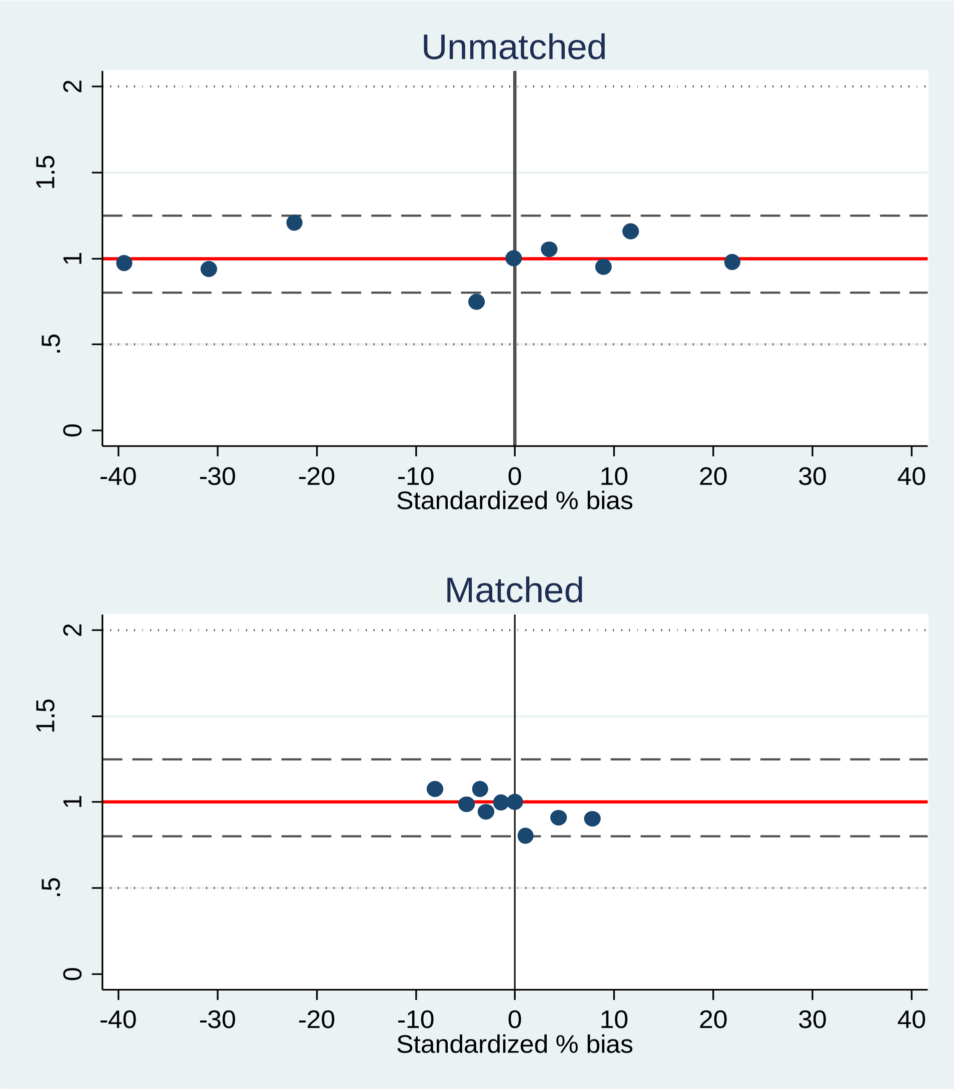

Standardised % bias is the % difference of the sample means in the treated and non-treated (full or matched) sub-samples as a percentage of the square root of the average of the sample variances in the treated and non-treated groups (formulae from Rosenbaum and Rubin, 1985).[1]

Graph 2. Standardized % bias across covariates

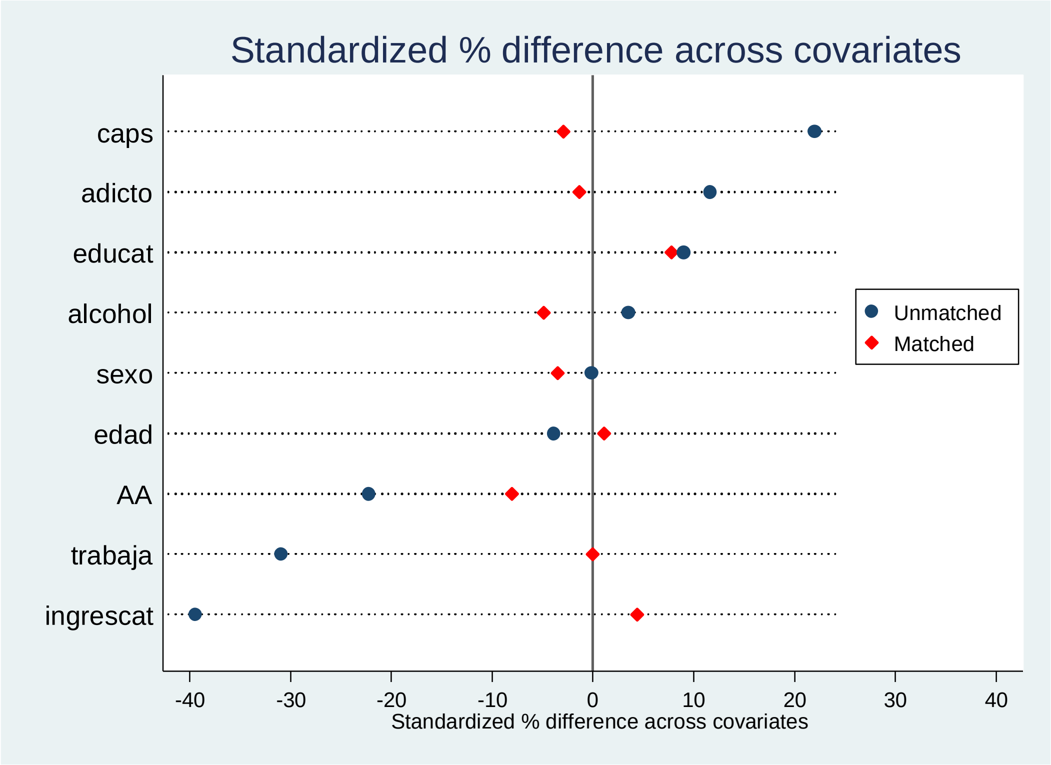

Comparing the similarity of treated and untreated subjects in the matched sample should begin with a comparison of the means or medians of continuous covariates and the distribution of their categorical counterparts between treated and untreated subjects.

The standardized difference can be used to compare the mean of continuous and binary variables between treatment groups. Although there is no universally agreed upon criterion as to what threshold of the standardized difference can be used to indicate important imbalance, a standard difference that is less than 0.1 (or 10%) has been taken to indicate a negligible difference in the mean or prevalence of a covariate between treatment groups.[2]

For a continuous covariate, the standardized difference is defined as

$$d = \frac{(\overline{x}_{treatment} - \overline{x}_{control})}{\sqrt{\frac{s^2_{treatment} + s^2_{control}}{2}}},$$

where *xtreatment* and *xcontrol* denote the sample mean of the covariate in treated and untreated subjects, respectively, whereas s2 treatment and s2 control denote the sample variance of the covariate in treated and untreated subjects, respectively.

For dichotomous variables, the standardized difference is defined as

$$d = \frac{(\hat{p}_{treatment} - \hat{p}_{control})}{\sqrt{\frac{\hat{p}_{treatment}(1 - \hat{p}_{treatment}) + \hat{p}_{control}(1 - \hat{p}_{control})}{2}}}$$

where *p treatment* and *p control* denote the prevalence or mean of the dichotomous variable in treated and untreated subjects, respectively. The standardized difference compares the difference in means in units of the pooled standard deviation. Furthermore, it is not influenced by sample size and allows for the comparison of the relative balance of variables measured in different units.[3]

Estimation of Treatment effects with Propensity score matching and Inverse probability weighting regression adjustment (IPWRA)[4,5]

Outputs from STATA 13

Treatment effect on default

IPWRA

```
Treatment-effects estimation                    Number of obs      =       855
Estimator      : IPW regression adjustment
------------------------------------------------------------------------------
             |               Robust
     Default |      Coef.   Std. Err.      z    P>|z|     [95% Conf. Interval]
-------------+----------------------------------------------------------------
POmeans      |
         CCT |
          0  |   .2175416   .0191326    11.37   0.000     .1800424    .2550408
          1  |   .0967414   .0156028     6.20   0.000     .0661604    .1273223
------------------------------------------------------------------------------
ATE      CCT |
   (1 vs 0)  |  -.1208003   .0242852    -4.97   0.000    -.1683983   -.0732022
-------------+----------------------------------------------------------------
```

PS MATCHING

```
Treatment-effects estimation                    Number of obs      =       855
Estimator      : propensity-score matching      Matches: requested =         1
Outcome model  : matching                                      min =         1
Treatment model: logit                                         max =         1
------------------------------------------------------------------------------
             |              AI Robust
     Default |      Coef.   Std. Err.      z    P>|z|     [95% Conf. Interval]
-------------+----------------------------------------------------------------
ATE          |
         CCT |
   (1 vs 0)  |  -.1192982   .0262541    -4.54   0.000    -.1707552   -.0678412
------------------------------------------------------------------------------
```

Treatment effect on success

IPWRA

```
Treatment-effects estimation                   Number of obs      =        857
Estimator      : IPW regression adjustment
------------------------------------------------------------------------------
             |               Robust
     Success |      Coef.   Std. Err.      z    P>|z|     [95% Conf. Interval]
-------------+----------------------------------------------------------------
POmeans      |
         CCT |
          0  |   .6745917   .0214532    31.44   0.000     .6325441    .7166393
          1  |    .825425   .0231753    35.62   0.000     .7800023    .8708477
------------------------------------------------------------------------------
             |               Robust
     Success |      Coef.   Std. Err.      z    P>|z|     [95% Conf. Interval]
-------------+----------------------------------------------------------------
ATE          |
         CCT |
   (1 vs 0)  |   .1508333   .0308924     4.88   0.000     .0902853    .2113812
-------------+----------------------------------------------------------------
```

PS MATCHING

```
Treatment-effects estimation                   Number of obs      =        857
Estimator      : propensity-score matching     Matches: requested =          1
Outcome model  : matching                                     min =          1
Treatment model: logit                                        max =          1
------------------------------------------------------------------------------
             |              AI Robust
       Exito |      Coef.   Std. Err.      z    P>|z|     [95% Conf. Interval]
-------------+----------------------------------------------------------------
ATE          |
    subsidio |
   (1 vs 0)  |   .1726954   .0346711     4.98   0.000     .1047412    .2406497
------------------------------------------------------------------------------
```

References

1. Rosenbaum, P.R. and Rubin, D.B. (1985), "Constructing a Control Group Using Multivariate Matched Sampling Methods that Incorporate the Propensity Score", The American Statistician 39(1), 33-38.
2. Austin, P.C. (2009), "Balance Diagnostics for Comparing the Distribution of Baseline Covariates Between Treatment Groups in Propensity Score Matched Samples." Statistics in Medicine 28(25), 3083-3107.
3. Austin, P.C . An Introduction to Propensity Score Methods for Reducing the Effects of confounding in Observational Studies. Multivariate Behavioral Research, 46:399–424, 2011. Copyright © Taylor & Francis Group, LLC ISSN: 0027-3171 print/1532-7906 online. DOI: 10.1080/00273171.2011.568786

4. Austin, P.C., Stuart E.A. Moving towards best practice when using inverse probability of treatment weighting (IPTW) using the propensity score to estimate causal treatment effects in observational studies. *Statist. Med. 2015,* 34 3661–3679
5. Changyu S, Xiaochun L, et al Sensitivity analysis for causal inference using inverse probability weighting. Biom J. 2011 September ; 53(5): 822–837. doi:10.1002/bimj.201100042.
