# Supplementary material for: Evaluation of a social protection policy on tuberculosis treatment outcomes: A prospective cohort study
Source: PLoS Med. 2019 Apr 30;16(4):e1002788. doi: 10.1371/journal.pmed.1002788 (PMC6490910; doi:10.1371/journal.pmed.1002788)
Supplement: S1 Approval — IRB, Institutional Review Board. (PDF) [file pmed.1002788.s004.pdf]

APROBACIÓN DEL PROTOCOLO Nº 1564

MIEMBROS DEL COMITE

Coordinadora:
DRA. KARIN KOPITOWSKI
Médica

DR. CARLOS BURGER
Abogado

DR. ARTURO CAGIDE
Médico

DRA. PAULA CAMBIASO
Bioquimica

DR. LUIS CATOGGIO
Doctor en Medicina

DR .LEONARDO GARFI
Médico

DR. GUSTAVO IZBIZKY
Médico

DR. AUGUSTO PÉREZ
Médico

San Justo, 18 de noviembre de 2010.

El **C**omité de **É**tica de **P**rotocolos de **I**nvestigación* del Hospital Italiano de Buenos Aires ha evaluado el protocolo **CARACTERÍSTICAS DE LOS PACIENTES Y DEL SISTEMA ASOCIADOS AL TRATAMIENTO EXITOSO DE LA TUBERCULOSIS,** versión en idioma inglés, y su **CONSENTIMIENTO INFORMADO,** versión en idioma español; aprobando la realización del estudio a cargo del Dr. Fernando Rubinstein.---

(APROBADO EN REUNIÓN DEL 18 DE NOVIEMBRE DE 2010, SEGÚN CONSTA EN LIBRO DE ACTAS Nº 4, FOLIO 139)

**Este Comité funciona de acuerdo con las normativas establecidas por el I.C.H. (F.D.A., -EEUU, Comunidad Europea, Japón)*

IRB00003580

**El Consentimiento que se adjunta, con firma y sello de este Comité, es el que corresponderá fotocopiar para entregar a los pacientes enrolados en el Hospital Italiano.

IMPORTANTE[1]:
Los investigadores deberán presentar a este Comité los formularios adjuntos, en el caso que corresponda:
1- Copia de la aprobación del protocolo por la ANMAT
2- Comunicación de inicio de la investigación (reclutamiento del 1º paciente)
3- Informe sobre la marcha del protocolo dentro del año de inicio del mismo:
- ✓ El progreso del protocolo con los pacientes reclutados y datos parciales si los hubiese, una vez por año, (salvo que este Comité decida que el informe deba ser más frecuente).
- ✓ Los eventos adversos informados por el patrocinante o detectados por Uds. mismos.
- ✓ Otras modificaciones al protocolo no se podrán aplicar sin ser antes evaluadas por este Comité, salvo en casos de riesgo de vida para el paciente.
- ✓ En caso de suspensión del protocolo, la comunicación deberá ser inmediata.

4- Comunicación de finalización de la investigación

De este documento se emiten tres copias: una para el archivo del Comité otra para el investigador y otra para el patrocinante (en el caso que corresponda).

[1] Ver Plan de seguimiento de protocolo en página 3 de este documento

No se solicitaron modificaciones

No requiere aprobación de ANMAT

CEPI
COMITÉ DE ÉTICA DE PROTOCOLOS DE INVESTIGACIÓN

PLAN DE SEGUIMIENTO

El **C**omité de **É**tica de **P**rotocolos de **I**nvestigación del Hospital Italiano de Buenos Aires, de acuerdo con la disposición 6550/08 de ANMAT, vigente desde el 1 día hábil del año 2009 realizará un seguimiento de este protocolo de investigación, a través de dos mecanismos:

1. El centro deberá remitir, siempre y en el caso que corresponda, completando los formularios adjuntos a la aprobación

    a. El formulario de aprobación de ANMAT, con la fotocopia de la disposición correspondiente en el caso del protocolo y del centro, enmiendas y consentimientos informados.
    b. informe de inicio de la investigación, completando correctamente el formulario de incorporación del Primer paciente
    c. El formulario de Remito de droga o dispositivo, debidamente acompañado del remito de la droga / dispositivo
    d. Informes periódicos (cada 6 meses) e informe final, completando el formulario de este comité correctamente,
    e. Informando sobre las inspecciones a realizarse en el centro a este comité, con el formulario correspondiente
    f. Informe de desviaciones, en el formulario de este comité.
    g. informe final con el formulario de este comité debidamente completo
    h. informes de eventos adversos enviados al investigador en formato electrónico de acuerdo a normativa del mismo.

    En caso de que de la documentación arriba mencionada se desprenda un alto reclutamiento de pacientes, un alto número de eventos adversos, y numerosas desviaciones, se procederá a realizar monitoreo y/o al menos una vez mientras este dure revisando, además de la documentación arriba mencionada, las historias clínicas de los pacientes, los consentimientos informados y toda otra documentación pertinente.

2. Y, acorde con la Disposición de ANMAT 690/05, (Guía de Inspecciones a Investigadores Clínicos)
    a. Por involucrar el estudio Población vulnerable,
    b. Por la Fase de investigación,
    c. Por ser la Investigación con riesgo mayor,
    d. Criterios de Selección de Centro,
    e. Alto reclutamiento,
    f. Por bajo/alto número de reportes de seguridad;
    g. Antecedentes del investigador;
    h. Elevado número de estudios por parte del investigador;
    i. Cualquier información relevante recibida en los reportes de seguridad, y/o en los informes de avance, que a criterio de este Comité amerite un monitoreo

Este Comité, por cumplir con las normas de GCP para actuar como Comité Independiente, no acepta ingerencia de otro comité independiente para este protocolo.

APROBACION DEL PROTOCOLO Nº 1564

MIEMBROS DEL COMITÉ

NOMBRE Y APELLIDO	CARGO	SEXO	FIRMA
Dr. Carlos **BURGER**	Abogado.<br>Ex Prof. Adjunto a cargo de la Cátedra de Bioética y Derecho.<br>Facultad de Derecho – UBA.<br>Prof. Jefe de Trabajos Prácticos – Facultad Medicina UBA	Masculino	
Dr. Arturo **CAGIDE**	Jefe del Servicio de Cardiología<br>Médico de Planta del Servicio de Cardiología	Masculino	
Bioq. Paula **CAMBIASO**	Investigadora.<br>Supervisora Laboratorios ICBME<br>Profesora Asistente de Inmunología Instituto Universitario Escuela de Medicina Hospital Italiano de Buenos Aires	Femenino	
Dr. Luis **CATOGGIO**	Médico de planta del Servicio de Clínica Médica Sección Reumatología<br>Profesor Asociado de Medicina<br>Instituto Universitario Escuela de Medicina, Hospital Italiano de Buenos Aires	Masculino	Ausente para la firma
Dr. Leonardo **GARFI**	Médico de planta del Servicio de Clínica Medica. Sección Farmacología Clínica<br>Prof. Adjunto del Dpto. de Farmacotoxicología<br>Instituto Universitario Escuela de Medicina, Hospital Italiano de Buenos Aires	Masculino	
Dr. Gustavo **IZBIZKY**	Subjefe del Servicio de Obstetricia<br>Médico de planta del Servicio de Obstetricia<br>Profesor Adjunto de Obstetricia - Instituto Universitario Escuela de Medicina, Hospital Italiano de Buenos Aires	Masculino	
Dra. Karin **KOPITOWSKI**	Médica de planta del Servicio de Medicina Familiar y Comunitaria.<br>Profesora titular de Medicina Familiar - Instituto Universitario Escuela de Medicina, Hospital Italiano de Buenos Aires	Femenino	
Dr. Augusto **PÉREZ**	Subjefe Unidad de Cuidados Intensivos Pediátricos<br>Médico de Planta Unidad de Cuidados Intensivos Pediátricos	Masculino	
