# Supplementary material for: Evaluation of a social protection policy on tuberculosis treatment outcomes: A prospective cohort study
Source: PLoS Med. 2019 Apr 30;16(4):e1002788. doi: 10.1371/journal.pmed.1002788 (PMC6490910; doi:10.1371/journal.pmed.1002788)
Supplement: S2 Approval — IRB, Institutional Review Board. (PDF) [file pmed.1002788.s005.pdf]

CEC
Comité de Ética Central

Ministerio de Salud Buenos Aires LA PROVINCIA

La Plata, Mayo 13 de 2.011

Sr Director Académico del I.E.C.S.

Dr. Fernando Rubinstein

S/D

NOTA CEC 040/2011

De mi mayor consideración:

Tengo el agrado de dirigirme a Ud. en referencia al estudio: **"Factores individuales y del sistema de salud asociados al tratamiento exitoso de la TBC"** Correspondiente al expediente 2919/183/2011.-

El Comité de Ética Central Informa que el estudio de referencia ha sido evaluado y aprobado por el Comité de Ética del Hospital Italiano sede San Justo, acreditado por el CEC y registrado ante la Comisión Conjunta de Investigación en Salud.-

En consecuencia en aquellos Centros que no cuenten con Comité de Ética e Investigación debidamente Acreditados según disposición del CEC, **se tome como válida** la aprobación realizada por el Comité de Ética del H. Italiano sede San Justo. Para completar el procedimiento se deberá obtener la aprobación de la autoridad máxima de la institución donde se realizará el estudio para permitir la subrogación.-

Sin otro en particular, saluda a Ud. muy atentamente

Dr. Juan Pablo Von Arx
Coordinador
Comité de Ética Central
