# Supplementary material for: Evaluation of a social protection policy on tuberculosis treatment outcomes: A prospective cohort study
Source: PLoS Med. 2019 Apr 30;16(4):e1002788. doi: 10.1371/journal.pmed.1002788 (PMC6490910; doi:10.1371/journal.pmed.1002788)
Supplement: S1 Appendix — CRF, case report form. (PDF) [file pmed.1002788.s006.pdf]

Identificación del paciente: ☐☐☐ ☐☐☐☐
Centro N° Paciente

B. Determinantes Socioeconómicos

1. Fecha de Nacimiento ☐☐ ☐☐ ☐☐☐☐ (DD/MM/AAAA)

2. Sexo: ☐ 1. Femenino ☐ 2. Masculino

3. Estado civil

☐ 1. Soltero ☐ 2. Casado ☐ 3. Divorciado ☐ 4.Separado ☐ 5. Viudo

4. Convive en pareja o matrimonio ☐ 1. Si ☐ 2. No

5. Nacionalidad

☐ 1. Argentina ☐ 2. Paraguaya ☐ 3. Uruguaya ☐ 4. Boliviana ☐ 5.Peruana

☐ 6. Chilena ☐ 7. Brasileña ☐ 8. Coreana ☐ 9. China ☐ 10. Otra

6. ¿Tiene residencia en Argentina mayor a 2 años?

☐ 1. Si **(Pase a la pregunta 7)** ☐ 2. No

6.a. ¿Hace cuánto tiempo que reside en la Argentina?

☐ 1. Hace menos de 6 meses ☐ 2. Entre 6 meses y 2 años

7. Habla Castellano: ☐ 1. Si ☐ 2. No

8. Sabe leer y escribir: ☐ 1. Si ☐ 2. No

9. Años de Educación Formal completados: ☐☐ años

10. ¿Actualmente está trabajando? ☐ 1. Si ☐ 2. No **(Pase a pregunta 11)**

10.a. ¿Qué tipo de trabajo es? ☐ 1. Formal ☐ 2. Informal

11. ¿Independientemente si trabaja o no trabaja, tiene alguna fuente de ingreso como jubilación/pensión/plan social/seguro?

☐ 1. Si ☐ 2. No **(Pase a pregunta 12)**

Identificación del paciente: ☐☐☐ ☐☐☐☐
Centro N° Paciente

11.a. ¿Qué tipo de fuente es?

☐ 1. Jubilación/ Pensión ☐ 2. Planes Sociales ☐ 3. Invalidez/seguro social ☐ 4. Otra

12. ¿Qué categoría describe mejor su ocupación laboral principal ?

☐ 1.Empleado ☐ 2. Profesional ☐ 3. Negocio/Cuenta propia

☐ 4. Oficio ☐ 5. Ama de casa ☐ 6. Changas

13. Tipo de Vivienda :

13.a ¿Tiene Vivienda? ☐ 1. Si ☐ 2. No **(Pase a pregunta 15)**

**13.b.**	**13c.**	**13d.**	**13e.**
☐ 1. Propia	☐ 1. Urbana	☐ 1. Unifamiliar	☐ 1. Construida
☐ 2. Alquilada	☐ 2. Semirural	☐ 2. Multifamiliar	☐ 2 Semiconstruida
☐ 3. Cedida	☐ 3. Rural		
☐ 4. Prestada	☐ 4. Bo. Emergencia		

13.f ¿Su vivienda, predominantemente, de que material es?

☐ 1. Ladrillo/Hormigón ☐ 2. Madera ☐ 3. Chapa ☐ 4. Carton ☐ 5. Otro

13.g. ¿Qué tipo de ventilación tiene la vivienda?

☐ 1. Ninguna ☐ 2. Sólo una ventana ☐ 3. Más de una ventana

13.h. ¿Cuántas habitaciones, que se usan como dormitorio, tiene la vivienda? ☐☐ Cantidad de habitaciones

13.i ¿ El paciente comparte las habitaciones con otras personas ?

☐ 1. Si ☐ 2. No **(Pase a pregunta 13.i)**

13.j ¿Con cuántas personas comparte la habitacion?

☐☐ **13.i.1** N° Menores ☐☐ **13.i.2** N° Mayores (mayor a 15 años)

13.k ¿El paciente comparte la cama con otra persona?

☐ 1. Si ☐ 2. No **(Pase a pregunta 14)**

Identificación del paciente: ☐☐☐ Centro N° ☐☐☐☐ Paciente

13.l ¿Con cuántas personas comparte la cama?

☐☐ **13.k.1** N° Menores ☐☐ **13.k.2** N° Mayores (mayor a 15 años)

14. ¿Cuántas personas conviven actualmente en su casa? **(Mínimo 6 meses de convivencia)**

☐☐ **14.1.** N° Menores ☐☐ **14.2.** N° Mayores (mayor a 15 años)

15. Composición del grupo familiar y/o cohabitantes

**Iniciales del apellido y nombre**	**Vínculo**	**Edad**	**Nacionalidad**

16. ¿Tiene cobertura de salud ? ☐ 1. Si ☐ 2. No **(Pase a pregunta 18)**

17. ¿Qué tipo de cobertura de salud tiene?:

☐ 1. Obra Social/PAMI ☐ 2. Prepaga ☐ 3. PROFE

18. ¿Cuál es la distancia que tiene hasta el Centro de Atención?:

☐ 1. Menor o igual a 10 cuadras ☐ 2. Mayor a 10 cuadras

19. ¿Tiene accesibilidad al centro de atención los días de lluvia? ☐ 1. Si ☐ 2. No

20. ¿En general concurre al centro?: ☐ 1. Solo ☐ 2. Acompañado

Identificación del paciente: ☐☐☐ Centro N° ☐☐☐☐ Paciente

21. ¿Me podría indicar en cúal de estos grupos se encuentra el ingreso mensual total del hogar?
(Incluya ingresos provenientes del trabajo, jubilaciones, changas, asignaciones familiares, seguro de desempleo, becas,

cuotas de alimentos, asignación por hijos, planes sociales y todas las otras formas de ingresos)

☐ 1. De $1 a $ 600 ☐ 4. De $2401 a $ 3550 ☐ 7. NS/NC

☐ 2. De $601 a $1200 ☐ 5. De $3551 a $ 4750

☐ 3. De $1201 a $ 2400 ☐ 6. De $4751 y más

C. Factores de riesgo

22. ¿Usted fuma o fumó cigarrillos?

☐ 1. Fumador actual ☐ 2. Ex. Fumador **(Pase a pregunta 23)** ☐ 3.Nunca **(Pase a pregunta 23)**

22a. ¿Cuántos? ☐☐☐ N° de Cigarrillos por día

22.b ¿ Cuantos años hace que fuma o fumo? ☐☐

23. ¿Tiene Diabetes**?** ☐ 1.Si ☐ 2. No **(Pase a pregunta 24)**

23.a ¿Está bajo tratamiento? ☐ 1.Si ☐ 2. No

24. ¿Recibe tratamiento inmunosupresor prolongado? ☐ 1. Si ☐ 2. No **(Pase a pregunta 25)**

24a. ¿Qué tipo de tratamiento recibe?

☐ 1. Oncológico ☐ 2. Insuficiencia Renal Crónica ☐ 3. Corticoides ☐ 4. Otro

25. ¿Tiene hepatopatía crónica? ☐ 1. Si ☐ 2. No

26. ¿Se realizó alguna vez el test de VIH? ☐ 1. Si ☐ 2. No **(Pase a pregunta 27)**

26a. ¿El resultado fue?

☐ 1. Positivo ☐ 2. Negativo **(Pase a pregunta 27)** ☐ 3. NS/NC **(Pase a pregunta 27)**

26a.1. ¿La carga viral fue < 50 copias?

☐ 1. Si ☐ 2. No ☐ 3. NS/NC

Identificación del paciente: ☐☐☐ Centro N° ☐☐☐☐ Paciente

26a.2. ¿Cuál fue el valor de CD4?? ☐☐☐☐ ☐ 1. NS/NC

27. ¿Consume o consumió algún tipo de droga, por un período mayor a 6 meses**?**

☐ 1. Si, actualmente ☐ 2. Si, en el pasado ☐ 3. No, nunca

28. ¿Toma algún tipo de bebida alcohólica?

☐ 1. Si, actualmente ☐ 2. Si, en el pasado ☐ 3. No, nunca **(Pase a pregunta 30)**

28a. ¿Qué tipo de bebida alcohólica toma? **(en litros)**

☐ 1.Vino	☐,☐ **28.a.1** Cantidad por día (L)	☐ **28.b.1** Días de la semana
☐ 2. Cerveza	☐,☐ **28.a.2** Cantidad por día (L)	☐ **28.b.2** Días de la semana
☐ 3. Bebida blanca	☐,☐ **28.a.3** Cantidad por día (L)	☐ **28.b.3** Días de la semana
☐ 4. Otra	☐,☐ **28.a.4** Cantidad por día (L)	☐ **28.b.4** Días de la semana

Cuestionario de Cage (pregunte textualmente al paciente)

29a. ¿Ha pensado en alguna ocasión que tenía que reducir o dejar la bebida?

☐ 1.Si ☐ 2. No

29b. ¿Le han molestado las observaciones de su familia, allegados, o conocidos acerca de sus hábitos de bebida?

☐ 1. Si ☐ 2. No

29c. ¿Se ha sentido alguna vez a disgusto o culpable, por su manera de beber?

☐ 1. Si ☐ 2. No

29d. ¿Alguna vez ha tenido que beber a primera hora de la mañana, para sentirse bien y empezar en forma el día?

☐ 1.Si ☐ 2. No

30. Con respecto al subsidio marque lo que tenga disponible el paciente

30a. ¿Tiene DNI Argentino? ☐ 1.Si ☐ 2. No

30b. ¿Tiene residencia en Argentina mayor a 2 años? ☐ 1.Si ☐ 2. No

Identificación del paciente: ☐☐☐ ☐☐☐☐

Centro N° Paciente

30c. ¿Tiene Baciloscopía Positiva? ☐ 1.Si ☐ 2. No

30d. ¿Está en condiciones de recibir el Subsidio? ☐ 1.Si ☐ 2. No

D. Enfermedad Actual

Definición de Categoría 1 "Caso nuevo": Paciente que nunca ha tomado drogas antituberculosas o que las

ha tomado por menos de un mes y que presenta:

— Tuberculosis pulmonar con baciloscopía positiva.

— Tuberculosis pulmonar con baciloscopía negativa (cultivo positivo o negativo), lesiones radiológicas extensas y sintomatología importante.

— Tuberculosis diseminada (miliar).

—Tuberculosis en pacientes con VIH/Sida.

31. Motivo de Consulta:

☐ 1. Sintomático Respiratorio ☐ 2. Contacto ☐ 3. Examen de salud ☐ 4. Otros

32. Fecha de Diagnóstico ☐☐ ☐☐ ☐☐☐☐ (DD/MM/AAAA)

33. Examen de Diagnóstico:

	Fecha: DD/MM/AAAA	Resultado
**Bacteriología**	**33.a.1:** / /	**33.a.2** ☐ 1. Positivo ☐ 2. Negativo ☐ 3. No realizado
**Rayos X**	**33.b.1:** / /	**33.b.2:** ☐ 1. Positivo ☐ 2. Negativo ☐ 3. No realizado
**PPD**	**33.c.1:** / /	**33.c.2:** ☐ 1. Positivo ☐ 2. Negativo ☐ 3. No realizado

34. Antecedentes de BCG: ☐ 1. Si ☐ 2. No ☐ 3. NS/NC

35. ¿Tiene localización extrapulmonar? ☐ 1. Si ☐ 2.No

36. Peso al inicio del tratamiento*:* ☐☐☐ Kg

37. ¿Sabe si alguien cercano a Ud. tiene o se encuentra en tratamiento para TBC? ☐ 1. Si ☐ 2.No

E. Tratamiento en la Fase Inicial

38. Fecha de Comienzo del Tratamiento: ☐☐ ☐☐ ☐☐☐☐ (DD/MM/AAAA)

39. Fecha estimada de finalización del tratamiento: ☐☐ ☐☐ ☐☐☐☐ (DD/MM/AAAA)

Identificación del paciente: ☐☐☐ ☐☐☐☐

Centro N° Paciente

40. Esquema de tratamiento elegido (marque con una cruz)

Medicamentos							
HRZE	HRZ	HR	H	R	Z	E	S

41. Modalidad del esquema elegido ☐ 1. Supervisado diario (TDO) ☐ 2. Autoadministrado

42. Fecha en que se completó el formulario: ☐☐ ☐☐ ☐☐☐☐ (DD/MM/AAAA)

43. Persona que completó el formulario:

____

Fecha: [|] [|] [2|0|1|] (DD/MM/AAAA)

Firma del Coordinador:
